# Steroidal Saponins from the Roots and Rhizomes of *Tupistra chinensis*

**DOI:** 10.3390/molecules200813659

**Published:** 2015-07-28

**Authors:** Yuze Li, Xin Wang, Hao He, Dongdong Zhang, Yi Jiang, Xinjie Yang, Fei Wang, Zhishu Tang, Xiaomei Song, Zhenggang Yue

**Affiliations:** 1 Shaanxi Collaborative Innovation Center of Chinese Medicinal Resource Industrialization, Shaanxi Province Key Laboratory of New Drugs and Chinese Medicine Foundation Research, Shaanxi Rheumatism and Tumor Center of TCM Engineering Technology Research, School of Pharmacy, Shaanxi University of Chinese Medicine, Xianyang 712046, China; E-Mails: lyz1990yeah@163.com (Y.L.); zhangnatprod@163.com (D.Z.); j9668216@126.com (Y.J.); xxx211xxx@126.com (X.Y.); wf88-88@163.com (F.W.); tzs6565@163.com (Z.T.); 2 The First Hospital of Xi’an, Xi’an 710002, China; E-Mail: wangxin.x@163.com; 3 School of Pharmaceutical Sciences, Xi’an Medical University, Xi’an 710021, China; E-Mail: hehao313@163.com

**Keywords:** *Tupistra chinensis*, steroidal saponins, structure identification, cytotoxic activity

## Abstract

Two new furostanol saponins **1**–**2** and a new spirostanol saponin **3** were isolated together with two known furostanol saponins **4**–**5** from the roots and rhizomes of *Tupistra chinensis*. Their structures were characterized as 1β,2β,3β,4β,5β,26-hexahydroxyfurost-20(22),25(27)-dien-5,26-*O*-β-d-glucopyranoside (**1**), 1β,2β,3β,4β,5β,6β,7α,23ξ,26-nona-hydroxyfurost-20(22),25(27)-dien-26-*O*-β-d-glucopyranoside (**2**), (20*S*,22*R*)-spirost-25 (27)-en-1β,3β,5β-trihydroxy-1-*O*-β-d-xyloside (**3**), tupisteroide B (**4**) and 5β-furost-Δ^25(27)^-en-1β,2β,3β,4β,5β,7α,22ξ,26-octahydroxy-6-one-26-*O*-β-d-glucopyranoside (**5**), respectively, by extensive use of spectroscopic techniques and chemical evidence. Additionally, the *in vitro* cytotoxic activity of **1**–**4** was evaluated on human A549 and H1299 tumor cell lines, and compound **3** exhibited cytotoxicity against A549 cells (IC_50_ 86.63 ± 2.33 μmol·L^−1^) and H1299 cells (IC_50_ 88.21 ± 1.34 μmol·L^−1^).

## 1. Introduction

**Figure 1:**
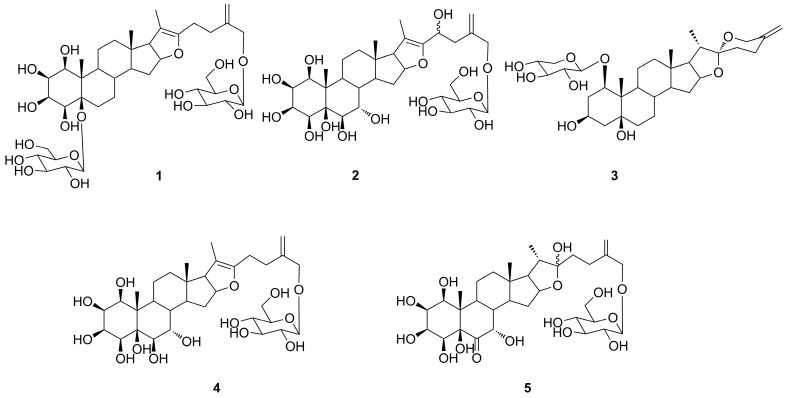
Structures of compounds **1**–**5**.

*Tupistra chinensis* Baker., a species in the *Tupistra* genus of the Liliaceae family, is used as an endemic herbal medicine, known as “Kai-Kou-Jian”, in the Qinba Mountains of Shaanxi Province in China [1]. The roots and rhizomes of *T.* c*hinensis* are commonly used as folk medicine to treat throat irritation, rheumatic diseases and snake-bites [2,3]. Modern pharmacological experiments have showed that the extracts of this species possess significant antitumor activities [4,5], moreover, two main kinds of components—cardenolides and saponins—were isolated from *T. chinensis* [3,6,7]. As part of our research project to find more diverse bioactive leading compounds from the medicinal herbs of the Qinba Mountains [8,9,10,11], the chemical constituents and pharmacological studies of *T. chinensis* were investigated, and two new furostanol saponins, 1β,2β,3β,4β,5β,26-hexahydroxyfurost-20(22),25(27)-dien-5,26-*O*-β-d-glucopyranoside (**1**), 1β,2β,3β,4β,5β,6β,7α,23ξ,26-nonahydroxyfurost-20(22),25(27)-dien-26-*O*-β-d-glucopyranoside (**2**), and a new spirostanol saponin (20*S*,22*R*)-spirost-25(27)-en-1β,3β,5β-trihydroxy-1-*O*-β-d-xyloside (**3**) were obtained from the roots and rhizomes of *T. chinensis* together with the two known compounds tupisteroide B (**4**) and 5β-furost-Δ^25(27)^-en-1β,2β,3β,4β,5β,7α,22ξ,26-octahydroxy-6-one-26-*O*-β-d-glucopyranoside (**5**) (Figure 1). The cytotoxic activity of **1**–**4** was evaluated on human A549 and H1299 tumor cells.

## 2. Results and Discussion

**Figure 2:**
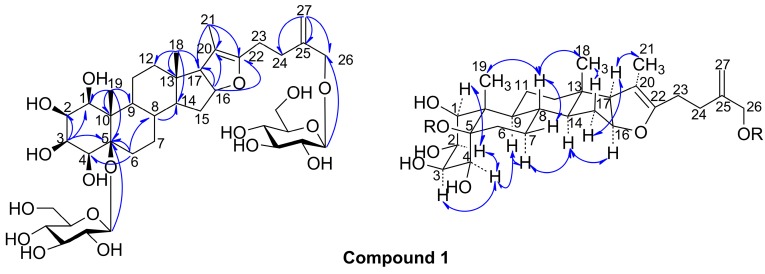
Key HMBC and NOESY correlations of the compound **1**.

Compound **1** was obtained as a white amorphous powder, which showed positive reactions in the Liebermann-Burchard, Ehrlich and Molisch reactions, suggesting that **1** was a furostanol glycoside. Its molecular formula was determined as C_39_H_62_O_17_ from the HR-ESI-MS peak at *m*/*z* 801.3855 [M − H]^−^. The ^1^H-NMR spectrum showed three methyl protons at δ_H_ 0.67 (3H, s), 1.70 (3H, s) and 1.58 (3H, s), two *exo*-methylene protons (δ_H_ 5.35 (1H, brs) and 5.04 (1H, brs)), as well as signals for two anomeric protons at (δ_H_ 5.28 (d, *J* = 7.8 Hz) and 4.89 (1H, d, *J* = 7.7 Hz)). The ^13^C-NMR spectrum displayed 39 carbon signals, 27 of which belonged to the aglycone carbons, while the remaining signals were assignable to two glucosyl moieties (δ_C_ 103.8, 75.8, 78.5, 71.7, 78.6 and 62.6, and δ_C_ 97.4, 76.2, 78.6, 71.9, 78.8 and 62.8). Among carbon signals of the aglycone, δ_C_ 146.2 and 111.6 were due to an olefinic bond group, δ_C_ 14.3, 13.7 and 11.7 were due to three methyl groups, and δ_C_ 77.8, 68.1, 75.2, 67.6, 87.4, 84.4, 64.5 and 71.7 were due to eight oxygenated carbon groups, which indicated that **1** was a furostanol saponin with multiple hydroxyl groups. The structure of **1** was finally determined by analysis of its 2D NMR data (see Figure 2). The HMQC experiment allowed for the assignments of the proton and protonated carbon resonances in the NMR spectra of **1**. HMQC correlations of (δ_H_ 5.35 (H-27a) and 5.04 (H-27b)) to δ_C_ 111.6, showed the appearance of a terminal olefinic bond at C-27. Then, HMBC correlations of H-27/C-24, C-25 and C-26, H-24/C-22, C-23, C-25 and C-26, H-26/C-24, C-25 and C-27, indicated that the appearance of an isopentene group, linked at C-22 of the tetrahydrofuran ring of the furostanol saponin. Moreover, HMBC correlations of H-19/C-1, C-5, C-9 and C-10, H-3/C-1, C-2 and C-5, and H-6/C-4 and C-5, indicated that all hydroxyl groups were linked at C-1–C-5 of the A ring of the furostanol saponin (see Figure 2). Furthermore, the remaining HMBC correlations of H-18/C-12, C-13, C-14 and C-17, H-16/C-13, C-17, C-20 and C-22, H-21/C-17, C-20 and C-22, were assigned (see Figure 2). Therefore, the aglycone of **1** was identified as 1, 2, 3, 4, 5, 26-hexanol-furost-20 (22),25(27)-dien. In addition, the HMBC correlation signals of H-Glc-1′/C-5 and H-Glc-1′′/C-26, indicated that glucosyl groups were connected as (Glc-1′′-*O*-C-26) and (Glc-1′-*O*-C-5) (see Figure 2). The two glucosyl moieties were identified as d-glucose by acid hydrolysis of **1**, followed by TLC comparison with a reference compound and optical rotation determination [12], and judged to be in a β-configuration [13] from the coupling constants of the anomeric protons (7.8 Hz and 7.7 Hz, respectively). In the NOESY spectrum of **1**, the NOE correlations of Me-19/H-8, H-9/H-4, H-4/H-3 and H-2, and H-2/H-1 were observed (see Figure 2), indicated α-axial configurations of H-1, H-2, H-3 and H-4, and β-orientation of Me-19, 1-OH, 2-OH, 3-OH, 4-OH and 5-OH, which supported the A/B *cis* ring junction pattern; the NOE correlations of Me-19/H-8, H-8/Me-18, and H-14/H-9, H-16 and H-17, supported the B/C and C/D *trans* ring junction pattern; and the NOE correlations of Me-18/H-15b, H-15a/H-16 and H-17, and H-17/Me-21, suggested an α-orientation of Me-21 (see Figure 2). Therefore, compound **1** was identified as 1β,2β,3β,4β,5β,26-hexahydroxyfurost-20(22),25(27)-dien-5,26-*O*-β-d-glucopyranoside.

**Figure 3:**
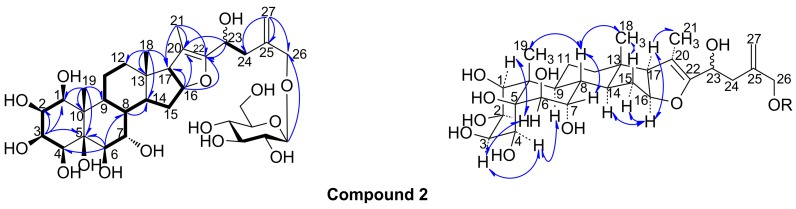
Key HMBC, ^1^H-^1^H COSY and NOESY correlations of the compound **2**.

Compound **2** was obtained as a white amorphous powder, which showed positive reactions in the Liebermann-Burchard, Ehrlich, and Molisch tests, suggesting that **2** was a furostanol glycoside. The molecular formula C_33_H_52_O_15_ was deduced from the HR-ESI-MS peak at *m*/*z* 711.3198 [M + Na]^+^. Comparison of the HR-ESI-MS and NMR data of **2** and **1**, indicated almost similar NMR spectroscopic features, except for the number of oxygenated methine groups. In the ^13^C-NMR spectrum of **2**, only one glucosyl moiety (δ_C_ 104.2, 75.6, 80.0, 72.1, 79.0, 63.2) was recognized, however, nine oxygenated carbon groups of the aglycone at δ_C_ 79.1, 67.7, 76.1, 70.2, 78.6, 74.0, 72.5, 64.8 and 72.7 were identified. Meanwhile, the spectroscopic features of **2** were similar to those of tupisteroide B (4), indicating that seven hydroxyl groups were linked at C-1–C-7 of the furostanol saponin, which was confirmed by the ^1^H-^1^H COSY correlation of H-1/H-2/H-3/H-4 and H-6/H-7 and the HMBC correlation of H-19/C-1, C-5, C-9 and C-10, and H-6/C-4 and C-5 (see Figure 3). The 26-OH was connected with the glucosyl moiety from the correlation signals of H-Glc-1′/C-26 in the HMBC spectra (see Figure 3). The remaining hydroxyl group was deduced to be linked at C-23, from one oxygen-bearing methine signal occurring at δ_C_ 64.8 in **2**, instead of a methylene carbon (C-23) at δ_C_ 34.3 in **4**, which was correlated with a proton signal at δ_H_ 5.13 (dd, *J* = 6.0, 8.0 Hz, H-23) in the HMQC spectrum, and the correlation signals of H-23/H-24 in the ^1^H-^1^H COSY spectrum, the correlation signals of H-23/C-20, C-22, C-24 and C-25, H-24/C-22, C-23, C-25, C-26 and C-27, and H-27/C-24, C-25 and C-26 in the HMBC spectrum (see Figure 3). In addition, the glucosyl moiety was identified as β-d-glucose by the acid hydrolysis procedure and the coupling constant analysis of the anomeric proton (*J* = 7.8 Hz), according to the same protocol as that described for **1**. Thus, the planar structure of **2** was deduced as 1,2,3,4,5,6,7,23,26-nonanolfurost-20(22),25(27)-dien-26-*O*-β-d-glucose. In the NOESY spectrum of **2**, the NOE correlations of Me-19/H-8, H-4/H-2, H-3 and H-9, and H-2/H-1 were observed, indicating α-axial configurations of H-1, H-2, H-3, and H-4, and β-orientation of Me-19, 1-OH, 2-OH, 3-OH, 4-OH and 5-OH, which supported the A/B *cis* ring junction pattern (see Figure 3). Besides, NOE correlation of H-7/H-8 was observed and no correlation signals was occurred between Me-19/H-6, which indicated α-axial configuration of 7-OH and β-orientation of 6-OH (see Figure 3). Finally, the NOE correlations of H-8/Me-19 and Me-18, and H-14/H-16 and H-17, supported the B/C and C/D *trans* ring junction pattern; and the NOE correlations of Me-18/H-15b, H-15a/H-16 and H-17, and H-17/Me-21, suggested the α-orientation of Me-21 (see Figure 3). Therefore, compound **2** was identified as 1β,2β,3β,4β,5β,6β,7α,23ξ,26-nonahydroxyfurost-20(22),25(27)-dien-26-*O*-β-d-glucopyranoside.

**Figure 4:**
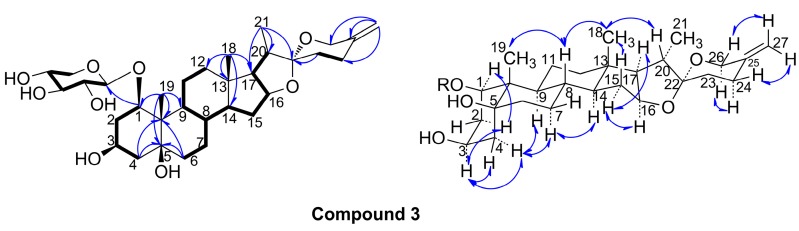
Key HMBC, ^1^H-^1^H COSY and NOESY correlations of the compound **3**.

Compound **3** was obtained as a white amorphous powder, and the molecular formula of C_32_H_50_O_9_ was established by the HR-ESI-MS signal at *m*/*z* 579.3590 [M + H]^+^. The ^13^C-NMR spectrum exhibited 32 carbon signals, 27 of which were attributed to the aglycone carbons, while the remaining signals were assignable to a characteristic of a xylosyl moiety (δ_C_ 104.1, 75.8, 78.9, 71.5 and 68.1), which was identified as β-d-xylose by the coupling constant analysis of the anomeric proton (*J* = 7.2 Hz), the acid hydrolysis procedure, TLC comparison, and the optical rotation determination. Among the aglycone carbon signals, the quaternary carbon signal at δ_C_ 109.9 (see, Table 1), was identified as an acetal carbon (C-22), a characteristic signal of spirostanol or norspirostanol saponin [14]. In HMBC spectrum, the anomeric proton [4.81 (1H, d, *J* = 7.2 Hz)] of the xylose was correlated with δ_C_ 82.5, which was confirmed as C-1 for the HMQC correlation of δ_H_ 4.26 (H-1)/δ_C_ 82.5 (C-1), ^1^H-^1^H COSY correlations of H-1/H-2/H-3/H-4, and HMBC correlations of H-19/C-1, C-5, C-9 and C-10 (see, Figure 4). Moreover, HMBC correlations of H-18/C-12, C-13, C-14 and C-17, H-21/C-17, C-20 and C-22, H-23/C-22, and H-27/C-24, C-25 and C-26, were observed (see, Figure 4).The above data indicated the planar structure of **3** as spirost-25(27)-en-1,3,5-trihydroxy-1-*O*-β-d-xyloside. In the NOESY spectrum of **3** (see, Figure 4), the NOE correlations of Me-19/H-8, H-3/H-2a and H-4, H-2a/H-1, and H-4a/H-7a and H-9, indicated α-axial configurations of H-1 and H-3, and β-orientation of Me-19, 1-OH, 3-OH and 5-OH, which supported the A/B *cis* ring junction pattern; the NOE correlations of H-8/Me-19 and Me-18, and H-14/H-9 and H-7a, supported the B/C and C/D *trans* ring junction pattern; the NOE correlations of Me-18/H-15b and H-20, H-15a/H-16 and H-17, and H-17/Me-21, suggested α-orientation of Me-21. These spectra data was almost similar to those of (20*S*,22*R*)-*1*β,3β,5β-trihydroxyspirost-25(27)-en-5-*O*-β-d-glucopyranoside [8], expect for the site of glycosylation. Therefore, compound **3** was elucidated as (20*S*, 22*R*)-spirost-25(27)-en-1β,3β,5β-trihydroxy-1-*O*-β-d-xyloside.

**Table 1:** ^1^H-NMR and ^13^C-NMR spectral data of compounds **1**–**3**.

Position	1		2		3	
	δc ^a^	δ_H_^a^ (*J* in Hz)	δc ^b^	δ_H_^b^ (*J* in Hz)	δc ^c^	δ_H_^c^ (*J* in Hz)
1	77.8	4.25 (brs)	79.1	4.29 (brs)	82.5	4.26 (brs)
2	68.1	4.38 (brs)	67.7	4.33 (brs)	30.4	2.53 (H-2a, *ca*.) 1.85 (H-2b, *ca*.)
3	75.2	4.70 (brs)	76.1	4.77 (brs)	67.8	4.59 (brs)
4	67.6	4.08 (brs)	70.2	5.33 (brs)	40.0	2.40 (H-4a, *ca*.) 2.04 (H-4b, *ca*.)
5	87.4	-	78.6	-	74.7	-
6	24.9	1.93 (*ca*.), 2.80 (*ca*.)	74.0	5.03 (brs)	36.3	1.54 (*ca*.), 1.90 (*ca*.)
7	28.5	1.1 (*ca*.), 1.51 (*ca*.)	72.5	4.49 (brs)	29.2	0.98 (H-7a, *ca*.) 1.51 (H-7b, *ca*.)
8	34.4	1.59 (*ca*.)	34.8	2.62 (*ca*.)	35.4	1.67 (*ca*.)
9	46.6	1.19 (*ca*.)	37.8	2.05 (*ca*.)	46.3	1.15 (*ca*.)
10	46.2	-	46.3	-	44.9	-
11	21.9	1.41 (*ca*.), 1.44 (*ca*.)	21.9	1.61 (*ca*.), 1.67 (*ca*.)	22.3	1.14(*ca*.),1.38 (*ca*.)
12	39.7	1.62 (d, 12.0), 1.15 (*ca*.)	40.0	1.70 (d, 12.0), 1.24 (*ca*.)	40.5	1.73 (d, 12.5), 1.13 (*ca*.)
13	43.3	-	43.8	-	41.2	-
14	54.3	0.76 (*ca*.)	48.9	1.96 (*ca*.)	56.7	1.12 (*ca*.)
15	31.0	2.48 (H-15a, *ca*.) 2.38 (H-15b, *ca*.)	34.7	2.58 (H-15a, *ca*.) 1.65 (H-15b, *ca*.)	32.7	2.07 (H-15a, *ca*.) 1.48 (H-15b, *ca*.)
16	84.4	4.77 (q, 7.5)	85.1	4.87 (*ca*.)	81.9	4.62 (q, 7.2)
17	64.5	2.42 (*ca*.)	65.3	2.57 (*ca*.)	63.5	1.88 (*ca*.)
18	14.3	0.67 (s)	14.6	0.81 (s)	17.0	0.87 (s)
19	13.7	1.70 (s)	16.0	1.99 (s)	14.4	1.59 (s)
20	103.9	-	105.9	-	42.4	2.00(*ca*.)
21	11.7	1.58 (s)	12.1	1.74 (s)	15.5	1.10 (d, 8.0)
22	151.8	-	154.2	-	109.9	-
23	34.3	1.45 (*ca*.), 2.04 (*ca*.)	64.8	5.13 (dd, 6.0, 8.0)	33.7	1.81 (*ca*.)
24	24.6	2.37 (*ca.*), 2.47 (*ca.*)	40.3	2.88 (H-24a, dd, 6.0, 14.3), 3.10 (H-24b, dd, 8.0, 14.3)	29.4	2.26 (*ca*.) 2.74 (*ca*.)
25	146.2	-	144.4	-	144.9	-
26	71.7	4.58 (d, 13.0) 4.34 (d, 13.0)	72.7	4.75 (d, 13.0) 4.61 (d, 13.0)	65.5	4.50 (d, 12.1) 4.07 (d, 12.1)
27	111.6	5.35 (H-27a, s) 5.04 (H-27b, s)	114.6	5.47 (H-27a, s) 5.28 (H-27b, s)	109.2	4.81(H-27a, s) 4.84 (H-27b, s)
1*'*	97.4	5.28 (d, 7.8)	104.2	5.0 (d, 7.8)	104.1	4.81 (d, 7.2)
2*'*	76.2	3.95 (*ca*.)	75.6	4.12 (*ca*.)	75.8	3.99 (*ca*.)
3*'*	78.6	4.01 (*ca*.)	80.0	4.36 (*ca*.)	78.9	4.21 (*ca*.)
4*'*	71.9	4.02 (*ca*.)	72.1	4.27 (*ca*.)	71.5	4.23 (*ca*.)
5*'*	78.8	4.22 (*ca*.)	79.0	3.96 (*ca*.)	68.1	3.78 (t, 10.5), 4.42 (dd, 4.5, 11.5)
6*'*	62.8	4.52 (*ca*.), 4.21 (*ca*.)	63.2	4.58 (dd, 2.0, 11.8), 4.41 (dd, 5.5, 11.8)	-	-
1*''*	103.8	4.89 (d, 7.7)	-	-	-	-
2*''*	75.8	4.03 (*ca*.)	-	-	-	-
3*''*	78.5	4.22 (*ca*.)	-	-	-	-
4*''*	71.7	4.19 (*ca*.)	-	-	-	-
5*''*	78.6	3.92 (*ca*.)	-	-	-	-
6*''*	62.6	4.52 (*ca.*), 4.35 (*ca.*)	-	-	-	-

^a^ δ in pyridine-*d*_5_, in ppm from TMS; coupling constants (*J*) in Hz; ^1^H-NMR at 500 MHz and ^13^C-NMR at 125 MHz; ^b^ δ in pyridine-*d*_5_, ^1^H-NMR at 600 MHz and ^13^C-NMR at 150 MHz; ^c^ δ in pyridine-*d*_5_, ^1^H-NMR at 400 MHz and ^13^C-NMR at 100 MHz.

Additionally, the known furostanol saponins were identified by comparison of their spectroscopic data with those reported in the literature as tupisteroide B (**4**) [15] and 5β-furost-Δ^25(27)^-en-1β,2β,3β,4β,5β,7α,22ξ,26-octaol-6-one-26-*O*-β-d-glucopyranoside (**5**) [16].

The cytotoxic activity of **1**–**4** towards the A549 and H1299 tumor cell lines was measured by the MTT method. Compound **3** exhibited cytotoxicity against A549 cells (IC_50_ 86.63 ± 2.33 μmol·L^−1^) and H1299 cells (IC_50_ 88.21 ± 1.34 μmol·L^−1^, see Table 2 and Table 3). Considering **3** is a spirostanol saponin, our results showed the cytotoxic activity of this type of steroidal saponin as mentioned in the literature [8,17,18,19].

**Table 2:** Activities of compounds **1**–**4** on proliferation of the H1299 cells.

Comp.	1 μM	3 μM	10 μM	30 μM	100 μM	IC_50_ μM
1	1.93 ± 0.95 **	13.50 ± 1.81 **	14.69 ± 1.41 **	16.53 ± 1.26 **	16.90 ± 0.69 **	>100
2	3.95 ± 2.09 **	5.75 ± 1.48 **	11.50 ± 3.22 **	16.17 ± 1.50 **	20.04 ± 1.36 **	>100
3	4.55 ± 1.10 **	8.04 ± 1.94 **	13.47 ± 0.61 **	17.39 ± 0.73 **	55.74 ± 0.87 **	88.21 ± 1.34
4	4.01 ± 0.86 **	9.26 ± 0.44 **	11.46 ± 2.91 **	13.47 ± 1.49 **	26.07 ± 0.99 **	>100
5-FU	3.07 ± 0.52	5.21 ± 0.28	17.39 ± 1.11	47.88 ± 1.38	71.96 ± 2.49	38.65 ± 1.59

The data are expressed as mean ± SD of three independent experiments (** *p* < 0.01 *vs.* control).

**Table 3:** Activities of compounds **1**–**4** on proliferation of the A549 cells.

Comp.	1 μM	3 μM	10 μM	30 μM	100 μM	IC_50_ μM
1	3.75 ± 1.24 **	11.62 ± 1.88 **	12.83 ± 2.02 **	14.35 ± 0.77 **	20.19 ± 3.63 **	>100
2	4.17 ± 1.30 **	7.68 ± 1.27 **	11.07 ± 1.57 **	13.80 ± 2.05 **	23.11 ± 0.74 **	>100
3	3.95 ± 0.95 **	7.90 ± 1.67 **	13.05 ± 1.75 **	20.60 ± 2.40 **	56.17 ± 1.98 **	86.63 ± 2.33
4	2.93 ± 1.18 **	6.65 ± 0.94 **	7.01 ± 2.47 **	13.21 ± 1.40 **	24.75 ± 1.62 **	>100
5-FU	6.97 ± 0.82	9.03 ± 1.21	23.76 ± 1.22	42.18 ± 1.22	69.24 ± 2.05	42.78 ± 1.63

The data are expressed as mean ± SD of three independent experiments (** *p* < 0.01 *vs.* control).

## 3. Experimental Section

### 3.1. General Information

The IR spectra were recorded on a TENSOR-27 instrument (Bruker, Rheinstetten, Germany). ESI-MS was performed on a Quattro Premier instrument (Waters, Milford, MA, USA). The HR-ESI-MS spectra were recorded on an Agilent Technologies 6550 Q-TOF (Santa Clara, CA, USA). 1D and 2D NMR spectra were recorded on Bruker-AVANCE 400, Bruker-AVANCE 500 and Bruker-AVANCE 600 instrument (Bruker, Rheinstetten, Germany) with TMS as an internal standard. The analytical HPLC was performed on a Waters 2695 Separations Module coupled with a 2996 Photodiode Array Detector and a Accurasil C18 column (4.6 mm × 250 mm, 5 mm particles, Ameritech, Chicago, IL, USA). Semipreparative HPLC was performed on a system comprising an LC-6AD pump (Shimadzu, Kyoto, Japan) equipped with a SPD-20A UV detector and a Ultimate XB-C18 (10 mm × 250 mm, 5 mm particles) or YMC-Pack-ODS-A (10 mm × 250 mm, 5 mm particles). D101 was from Sunresin New Materials Co. Ltd. (Xi’an, China). Silica gel was purchased from Qingdao Haiyang Chemical Group Corporation (Qingdao, China).

### 3.2. Plant Material

The roots and rhizomes of *T. chinensis* Baker were collected from the Taibai region of Qinba Mountains in Shaanxi Province, China, in August 2010, and identified by senior experimentalist Jitao Wang. A voucher specimen (herbarium No. 20100816) has been deposited in the Medicinal Plants Herbarium (MPH), Shaanxi University of Chinese Medicine, Xianyang, China.

### 3.3. Extraction and Isolation

The air-dried and powdered underground parts of *T. chinensis* (1.5 kg) were extracted with 65% EtOH (15 L) three times at 80 °C. The combined EtOH extracts were evaporated to 6 L, and applied to a resin D101 column, eluting with H_2_O, 20% EtOH, 60% EtOH, and 95% EtOH to give four fractions (Fr.1–Fr.4). Fr.3 (75 g) was subjected to column chromatography (CC) on silica gel, eluting with gradient solvent system (CHCl_3_–MeOH–H_2_O, 100:0:0–0:50:50) to yield nine fractions (Fr.3-1–Fr.3-9). Fr.3-6 (5 g) was separated over silica gel using CHCl_3_–MeOH (100:1–50:50) as eluent to obtain eight fractions (Fr.3-6-1–Fr.3-6-8). Fr.3-6-5 (150 mg) and Fr.3-6-7 (370 mg) were purified by HPLC (YMC-Pack-ODS-A, 10 mm × 250 mm, 5 μm particles, flow rate: 1.0 mL∙min^−1^) with CH_3_OH–H_2_O (45:55) as mobile phase to afford **1** (23 mg; *t_R_* = 35 min), **2** (15 mg; *t_R_* = 27 min), **3** (20 mg; *t_R_* = 43 min), **4** (27 mg; *t_R_* = 47 min) and **5** (1.8 mg; *t_R_* = 65 min).

### 3.4. 1β,2β,3β,4β,5β,26-Hexahydroxyfurost-20(22),25(27)-dien-5,26-O-β-d-glucopyranoside (**1**)

A white amorphous powder, IR (KBr) ν_max_: 3450, 2980, 1694, 1025, 907, 804, 772 cm^−1^. ^1^H-NMR (500 MHz, pyridine-*d*_5_) and ^13^C-NMR (125 MHz, pyridine-*d*_5_) spectral data, see Table 1; *m*/*z* 801.3855 [M − H]^−^ (calcd. for C_39_H_61_O_17_, 801.3909). 

### 3.5. 1β,2β,3β,4β,5β,6β,7α,23ξ,26-Nonahydroxyfurost-20(22),25(27)-dien-26-O-β-d-glucopyranoside (**2**)

A white amorphous powder, IR (KBr) ν_max_: 3475, 2980, 1742, 1062, 904, 804 cm^−1^. ^1^H-NMR (600 MHz, pyridine-*d*_5_) and ^13^C-NMR (150 MHz, pyridine-*d*_5_) spectral data, see Table 1; *m*/*z* 711.3198 [M + Na]^+^ (calcd. for C_33_H_5__2_O_15_Na, 711.3204).

### 3.6. (20S,22R)-Spirost-25(27)-en-1β,3β,5β-trihydroxy-1-O-β-d-xyloside (**3**)

A white amorphous powder; IR (KBr) ν_max_: 3306, 2922, 1650, 1042, 989, 917, 892, 876 cm^−1^; ^1^H-NMR (400 MHz, pyridine-*d*_5_) and ^13^C-NMR (100 MHz, pyridine-*d*_5_) spectral data, see Table 1; *m*/*z* 579.3590 [M + H]^+^ (calcd. for C_32_H_51_O_9_, 579.3633).

### 3.7. Acid Hydrolysis of Compounds **1**, **2**, **3** and Absolute Sugar Configuration Determination

The solutions of compounds **1** (3 mg), **2** (3 mg) and **3** (5 mg) were hydrolyzed with 2*N* HCl (5 mL) for 5 h at 80 °C, respectively. The reaction mixtures were concentrated and dried by N_2_, and then water (5 mL) was added and the mixtures were extracted with EtOAc (3 × 5 mL). The aqueous layers of **1** and **2** were subjected to CC over silica gel eluted with MeCN–H_2_O (8:1) to yield d-glucose, which was determined by TLC comparison (MeCN–H_2_O, 6:1) with the authentic sugar and the optical rotation determination ${\left[\alpha \right]}_{D}^{\mathrm{20}}$ +49.2 (*c* 0.16, H_2_O). The aqueous layer of **3** was subjected to CC over silica gel eluted with MeCN–H_2_O (8:1–15:1) to yield d-xylose, which was identified by TLC comparison with the authentic sugar and the optical rotation determination ${\left[\alpha \right]}_{D}^{\mathrm{20}}$ +17.9 (*c* 0.14, H_2_O).

### 3.8. Cytotoxicity Assay

The cytotoxic activity assays towards the A549 and H1299 tumor cell lines were measured by the MTT method *in vitro*, using 5-fluorouracil as positive control. Briefly, 1 × 10^4^ mL^−1^ cells were seeded into 96-well plates and allowed to adhere for 24 h. Compounds **1**–**4** were dissolved in DMSO and diluted with complete medium to five concentration levels (from 0.001 mmol·L^−1^ to 0.1 mmol·L^−1^) for inhibition rate determination. After incubation at 37 °C for 4 h, the supernatant was removed before adding DMSO (100 μL) to each well. 5-Fluorouracil (5-Fu) was used as positive control. The inhibition rate (IR) and IC_50_ were calculated. Values are mean ± SD, *n* = 3, ** *p* < 0.01 *vs.* DMEM control. Compound **3** exhibited cytotoxicity against A549 cells (IC_50_ 86.63 ± 2.33 μmol·L^−1^) and H1299 cells (IC_50_ 88.21 ± 1.34 μmol·L^−1^), while the positive control of 5-Fu exhibited cytotoxicity against A549 cells (IC_50_ 42.78 ± 1.63 μmol·L^−1^) and H1299 cells (IC_50_ 38.65 ± 1.59 μmol·L^−1^), respectively, (see Table 2 and Table 3).

## Supplementary Materials

IR, HR-ESI-MS, ^1^H-NMR ^13^C-NMR and 2D NMR spectra for compounds **1**–**3** can be accessed at: http://www.mdpi.com/1420-3049/20/08/13659/s1.
