# Supplementary material for: Steroidal Saponins from the Roots and Rhizomes of Tupistra chinensis
Source: Molecules. 2015 Jul 28;20(8):13659–69. doi: 10.3390/molecules200813659 (PMC6332035; doi:10.3390/molecules200813659)
Supplement: Supplementary file 1 [file molecules-20-13659-s001.pdf]

Supplementary Materials

List of Content

**No.**	**Content**	**Page**
1	**Figure S1.** The IR spectrum of **1** (in KBr)	S2
2	**Figure S2.** The HR-ESI-MS spectrum of **1** (in MeOH)	S3
3	**Figure S3.** The ^{1}H-NMR spectrum of **1** (in C_5D_5N)	S4
4	**Figure S4.** The ^{13}C-NMR spectra of **1** (in C_5D_5N)	S5
5	**Figure S5.** The HMQC spectrum of **1** (in C_5D_5N)	S6
6	**Figure S6.** The HMBC spectrum of **1** (in C_5D_5N)	S7
7	**Figure S7.** The ROESY spectrum of **1** (in C_5D_5N)	S8
8	**Figure S8.** The IR spectrum of **2** (in KBr)	S9
9	**Figure S9.** The HR-ESI-MS spectrum of **2**(in MeOH)	S10
10	**Figure S10.** The ^{1}H-NMR spectrum of **2** (in C_5D_5N)	S11
11	**Figure S11.** The ^{13}C-NMR spectra of **2** (in C_5D_5N)	S12
12	**Figure S12.** The HMQC spectrum of **2** (in C_5D_5N)	S13
13	**Figure S13.** The ^{1}H-^{1}H COSY spectrum of **2** (in C_5D_5N)	S14
14	**Figure S14.** The HMBC spectrum of **2** (in C_5D_5N)	S15
15	**Figure S15.** The NOESY spectrum of **2** (in C_5D_5N)	S16
16	**Figure S16.** The IR spectrum of **3** (in KBr)	S17
17	**Figure S17.** The HR-ESI-MS spectrum of **3**(in MeOH)	S18
18	**Figure S18.** The ^{1}H-NMR spectrum of **3** (in C_5D_5N)	S19
19	**Figure S19.** The ^{13}C-NMR spectra of **3** (in C_5D_5N)	S20
20	**Figure S20.** The HMQC spectrum of **3** (in C_5D_5N)	S21
21	**Figure S21.** The ^{1}H-^{1}H COSY spectrum of **3** (in C_5D_5N)	S22
22	**Figure S22.** The HMBC spectrum of **3** (in C_5D_5N)	S23
23	**Figure S23.** The NOESY spectrum of **3** (in C_5D_5N)	S24

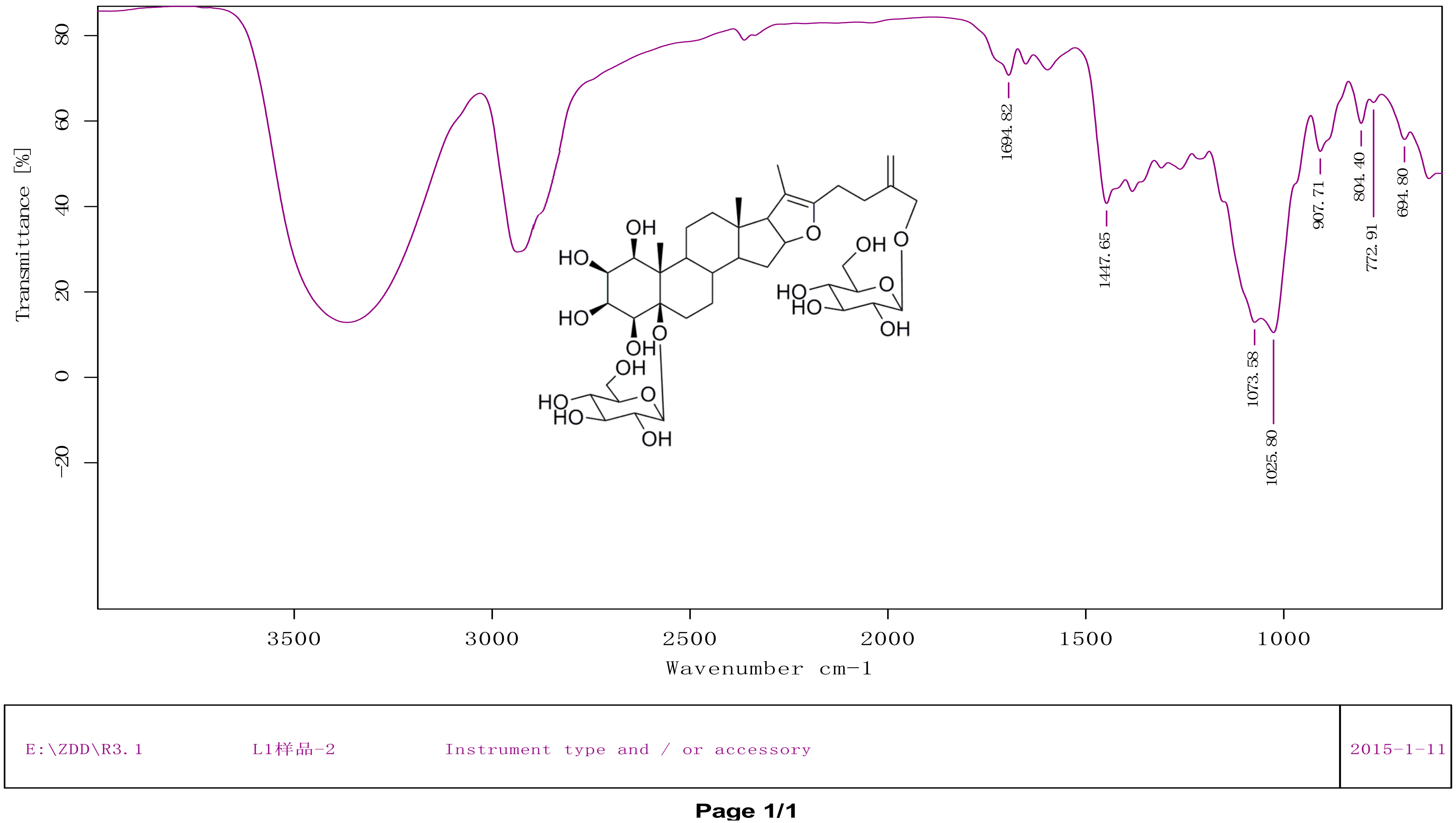

Figure S1. The IR spectrum of **1** (in KBr).

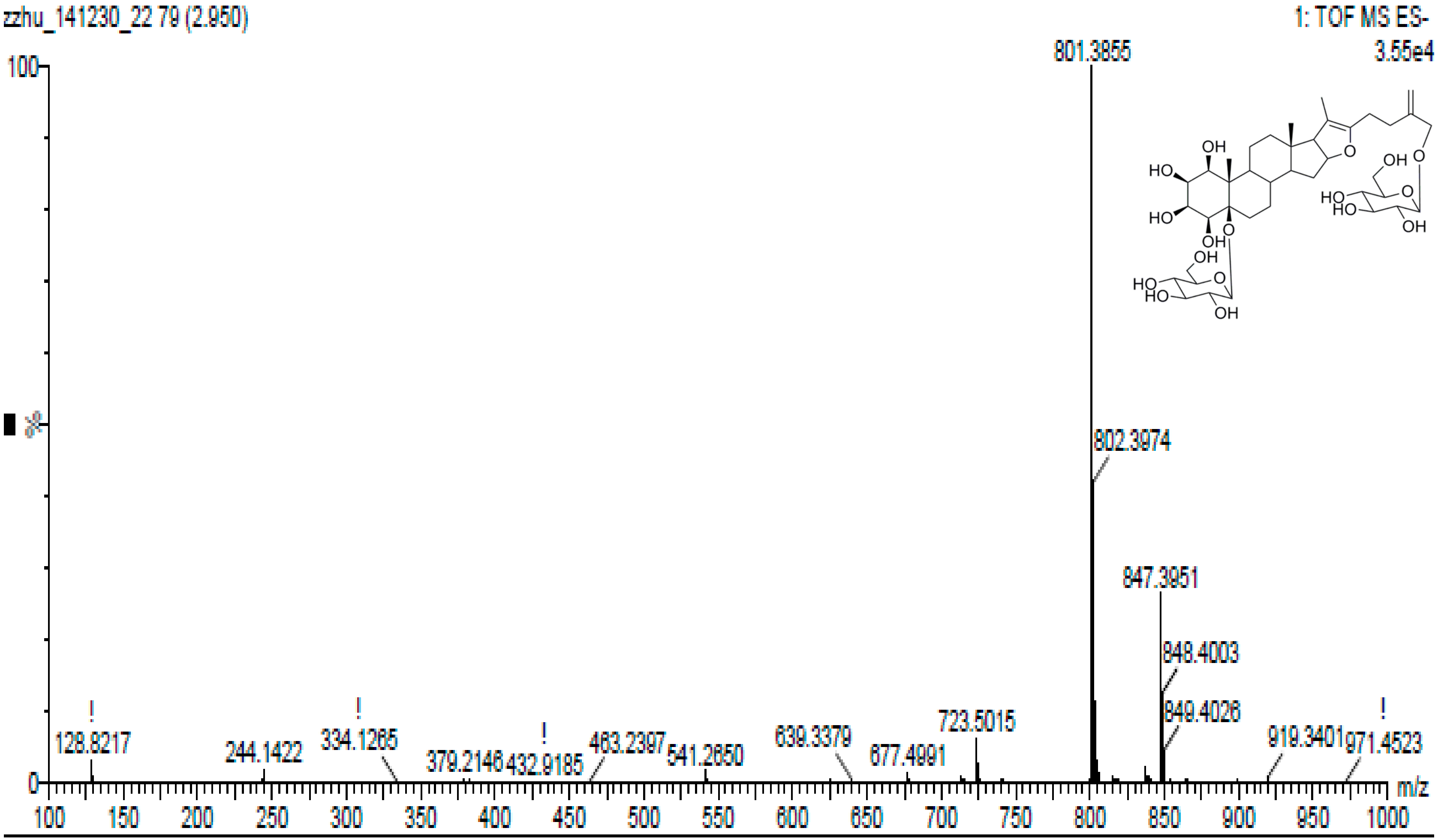

Figure S2. The HR-ESI-MS spectrum of **1** (in MeOH).

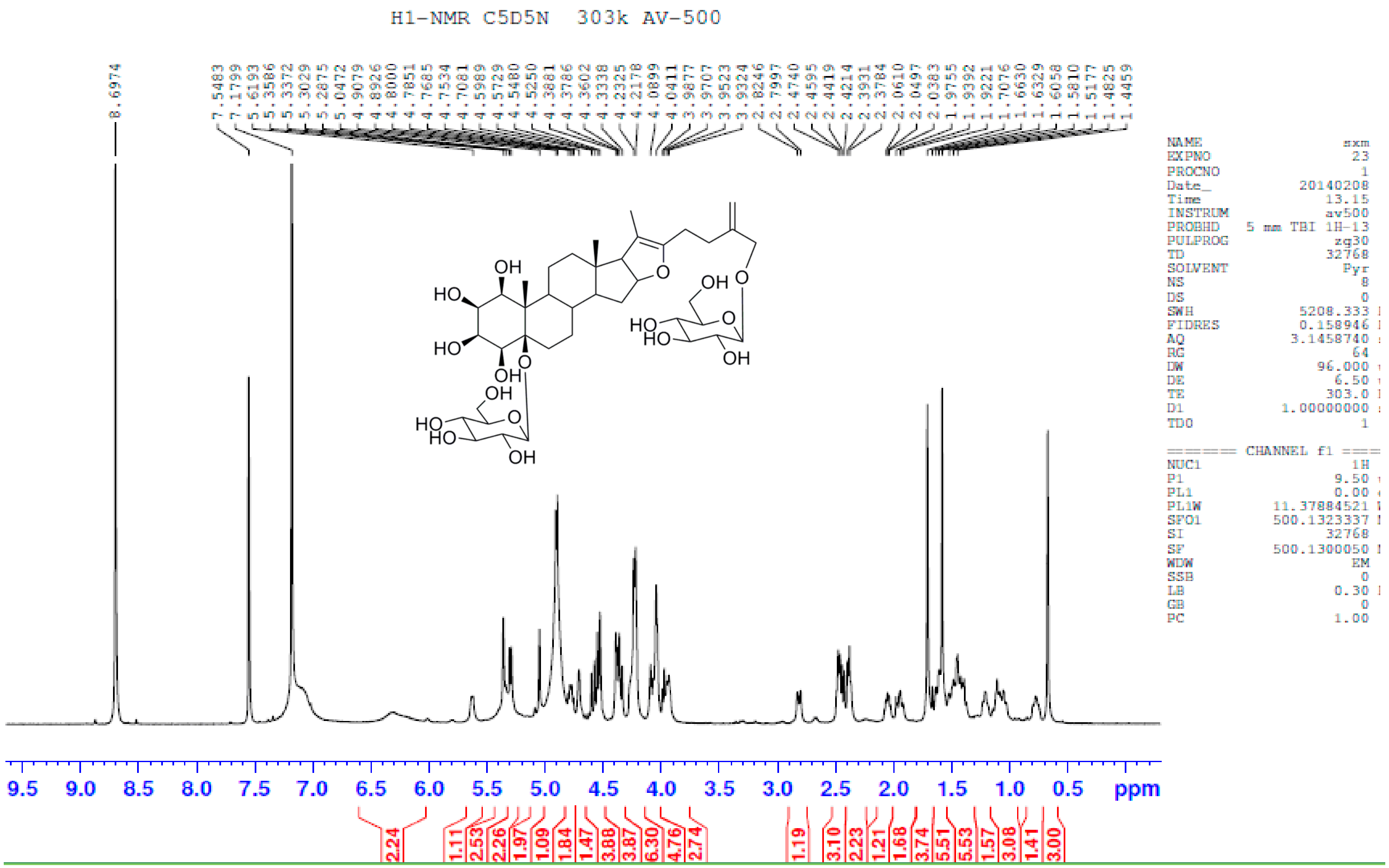

Figure S3. The ^{1}H-NMR spectrum of **1** (in C_5D_5N).

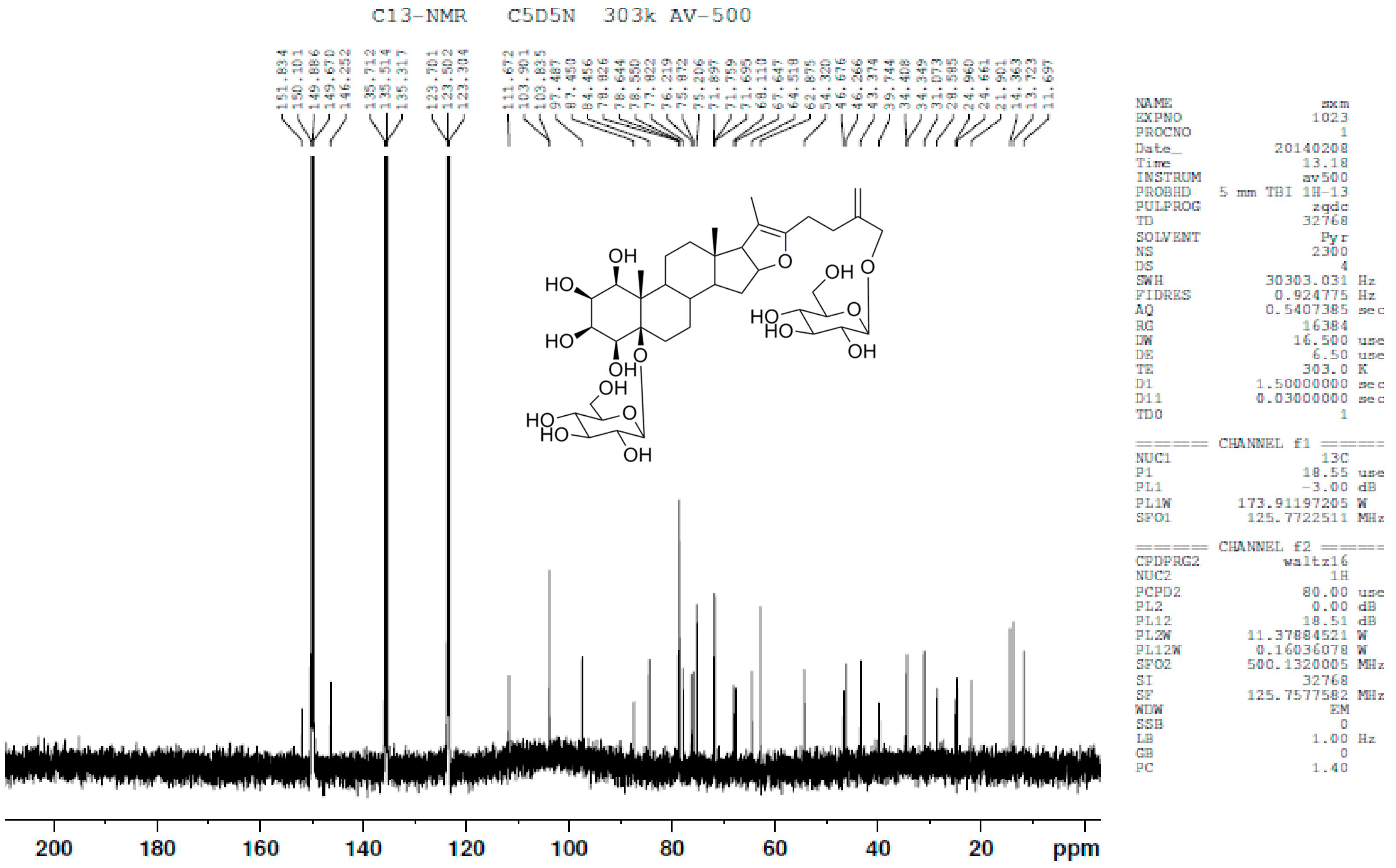

Figure S4. The ^{13}C-NMR spectrum of **1** (in C_5D_5N).

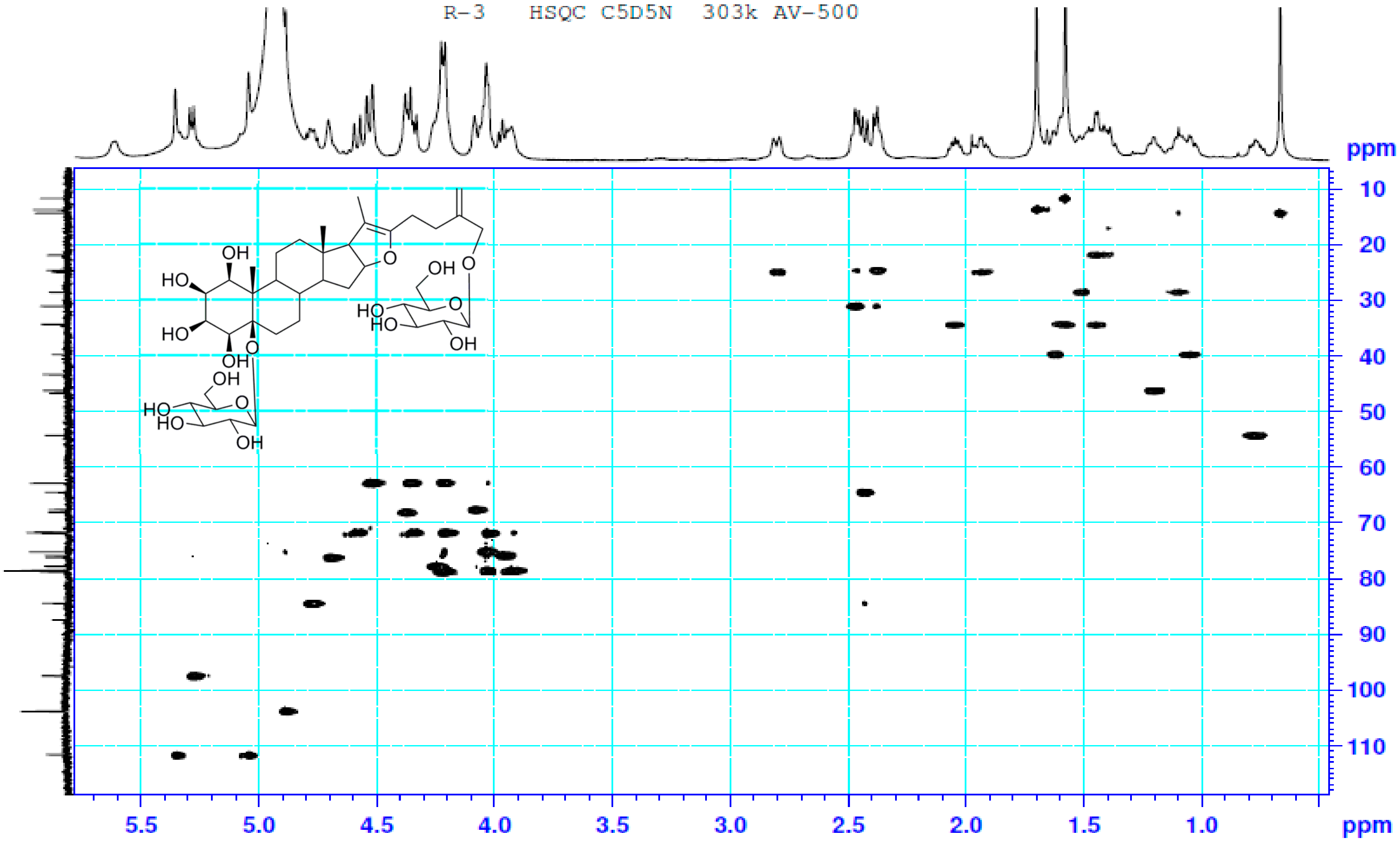

Figure S5. The HMQC spectrum of **1** (in C_5D_5N).

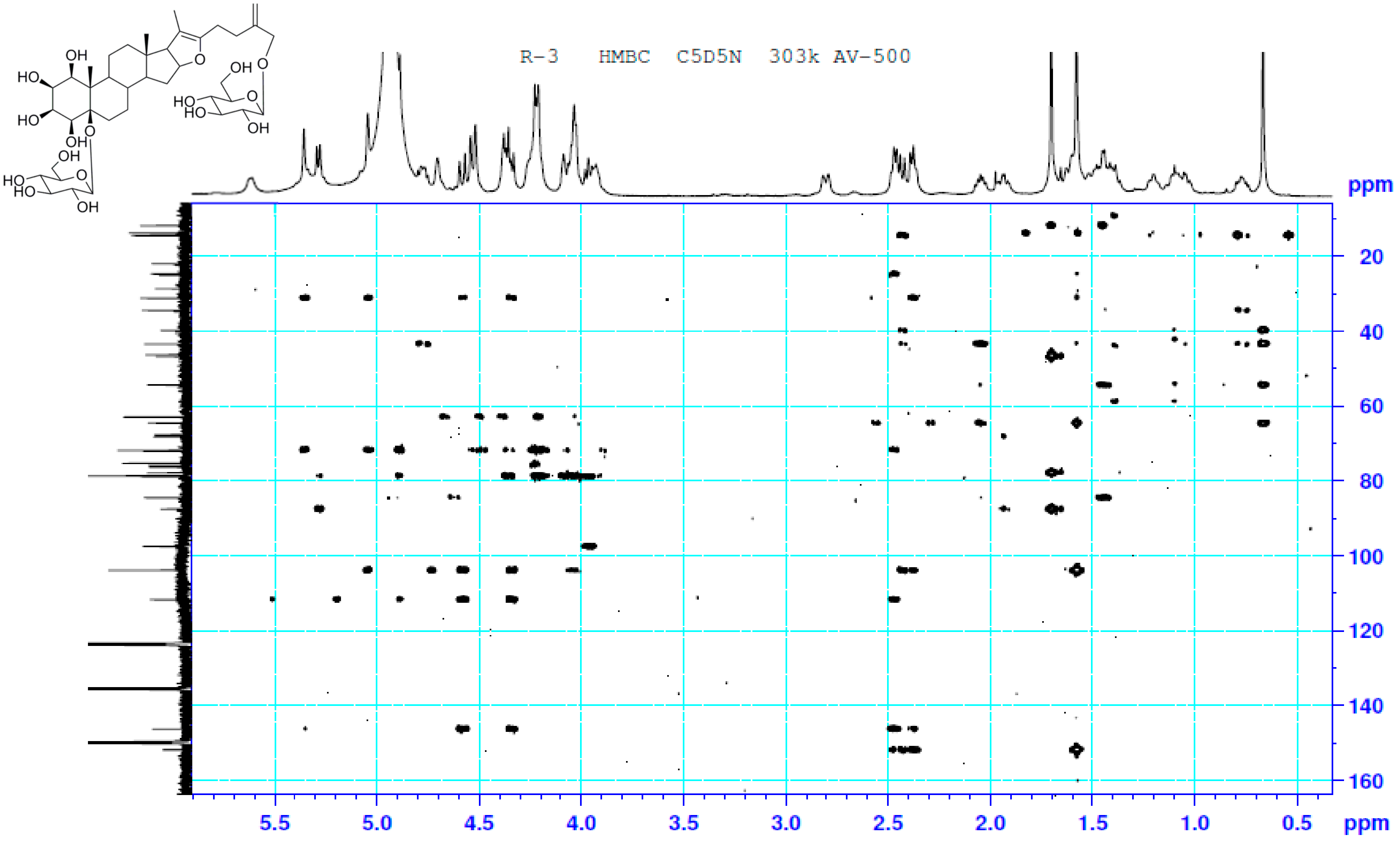

Figure S6. The HMBC spectrum of **1** (in C_5D_5N).

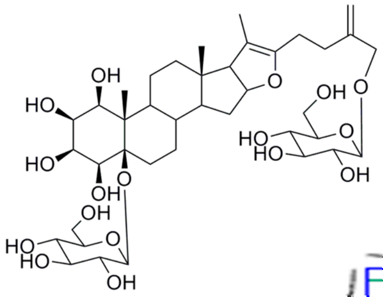

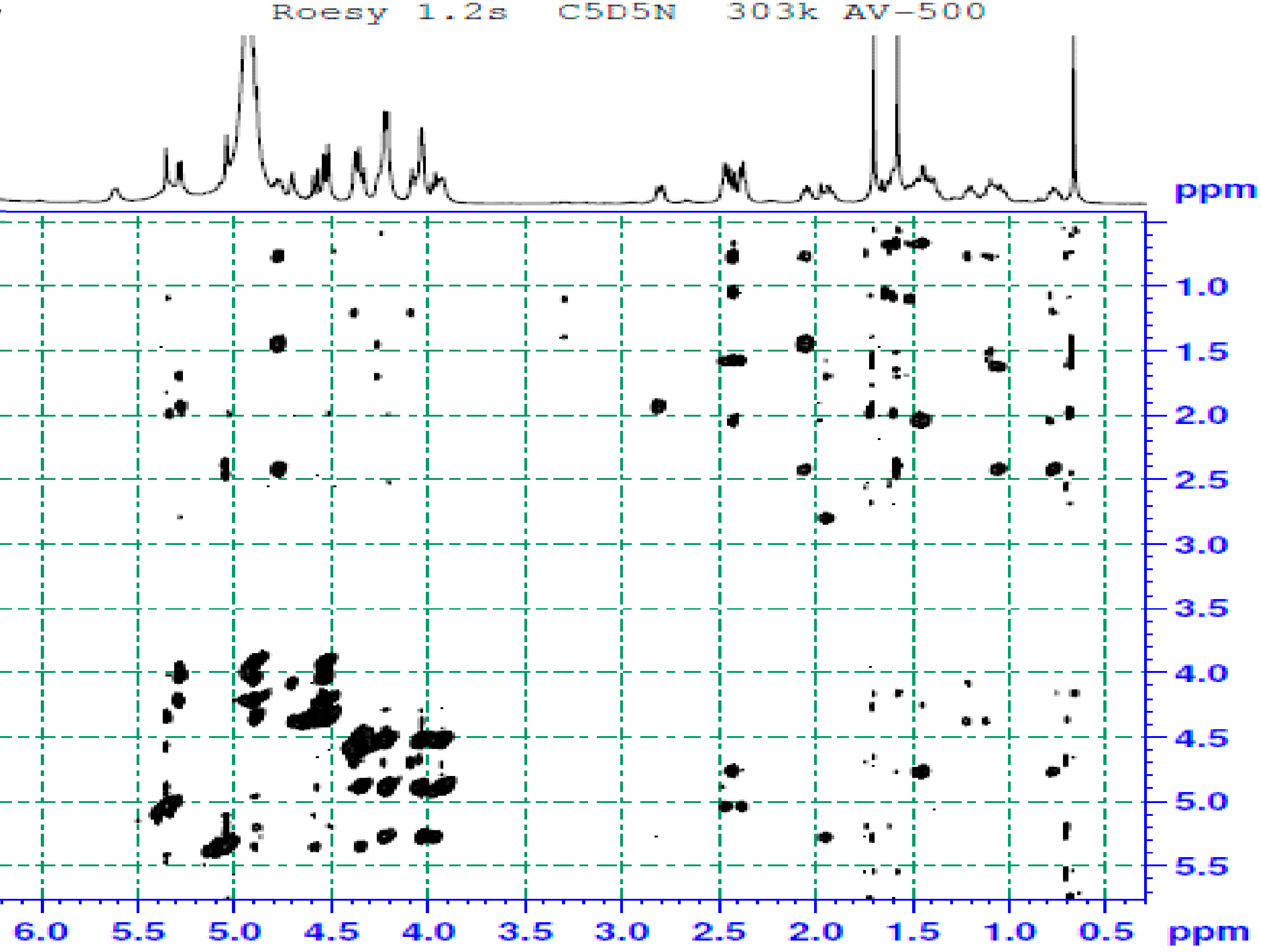

Figure S7. The ROESY spectrum of **1** (in C_5D_5N).

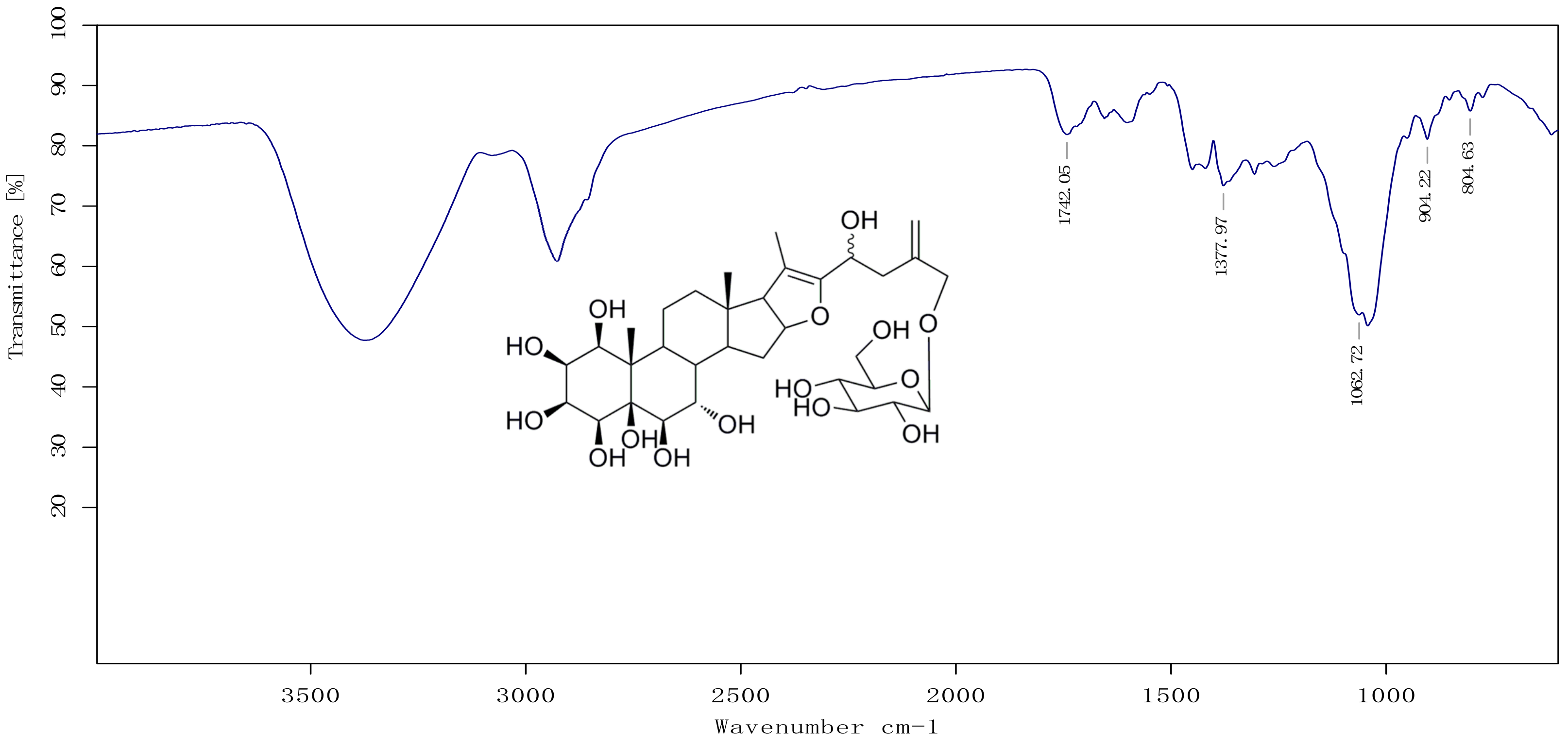

E:\ZDD\R22.1	L1样品-2	Instrument type and / or accessory	2015-1-7

Page 1/1

Figure S8. The IR spectrum of **2** (in KBr).

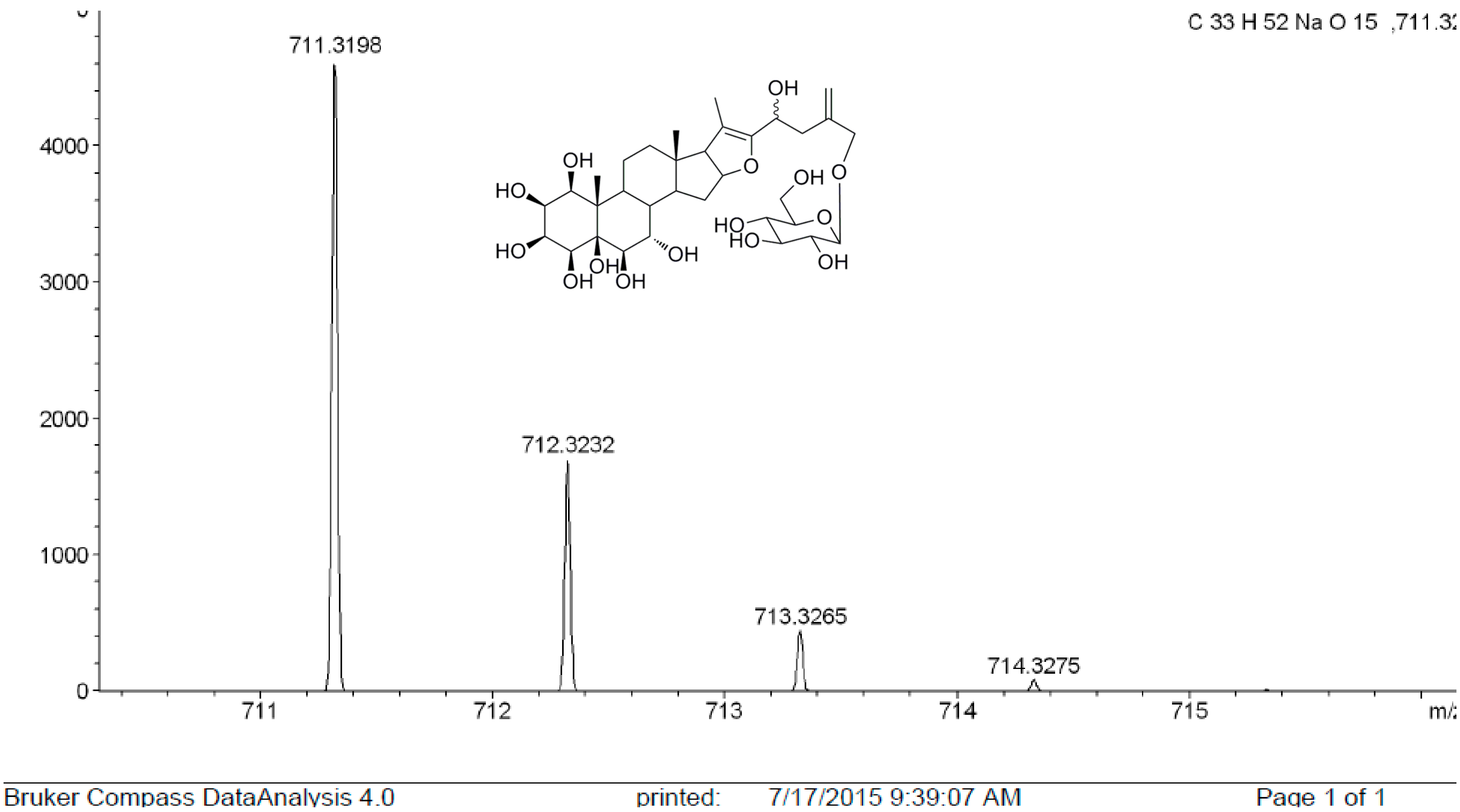

Figure S9. The HR-ESI-MS spectrum of **2** (in MeOH).

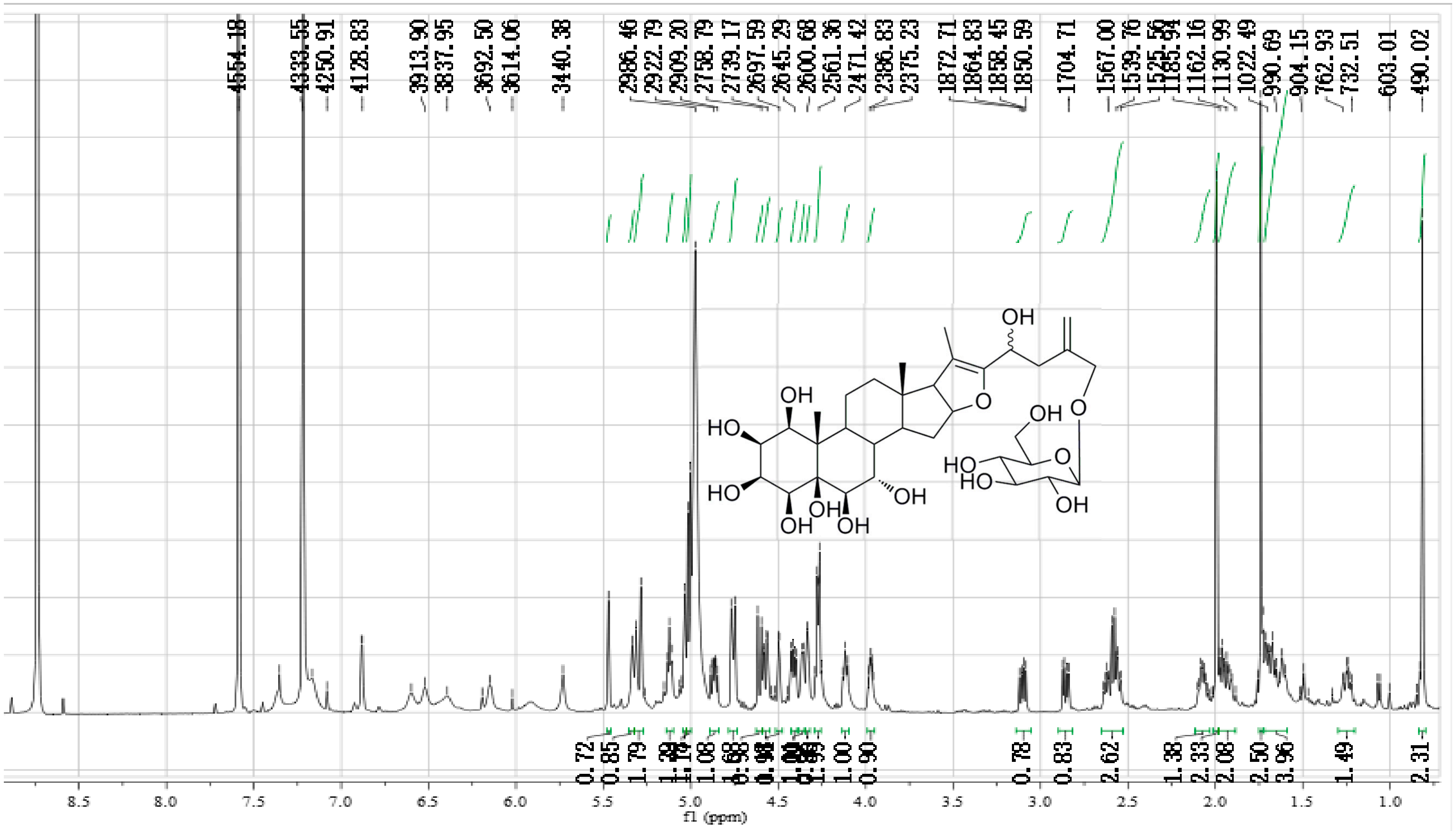

Figure S10. The ^{1}H-NMR spectrum of **2** (in C_5D_5N).

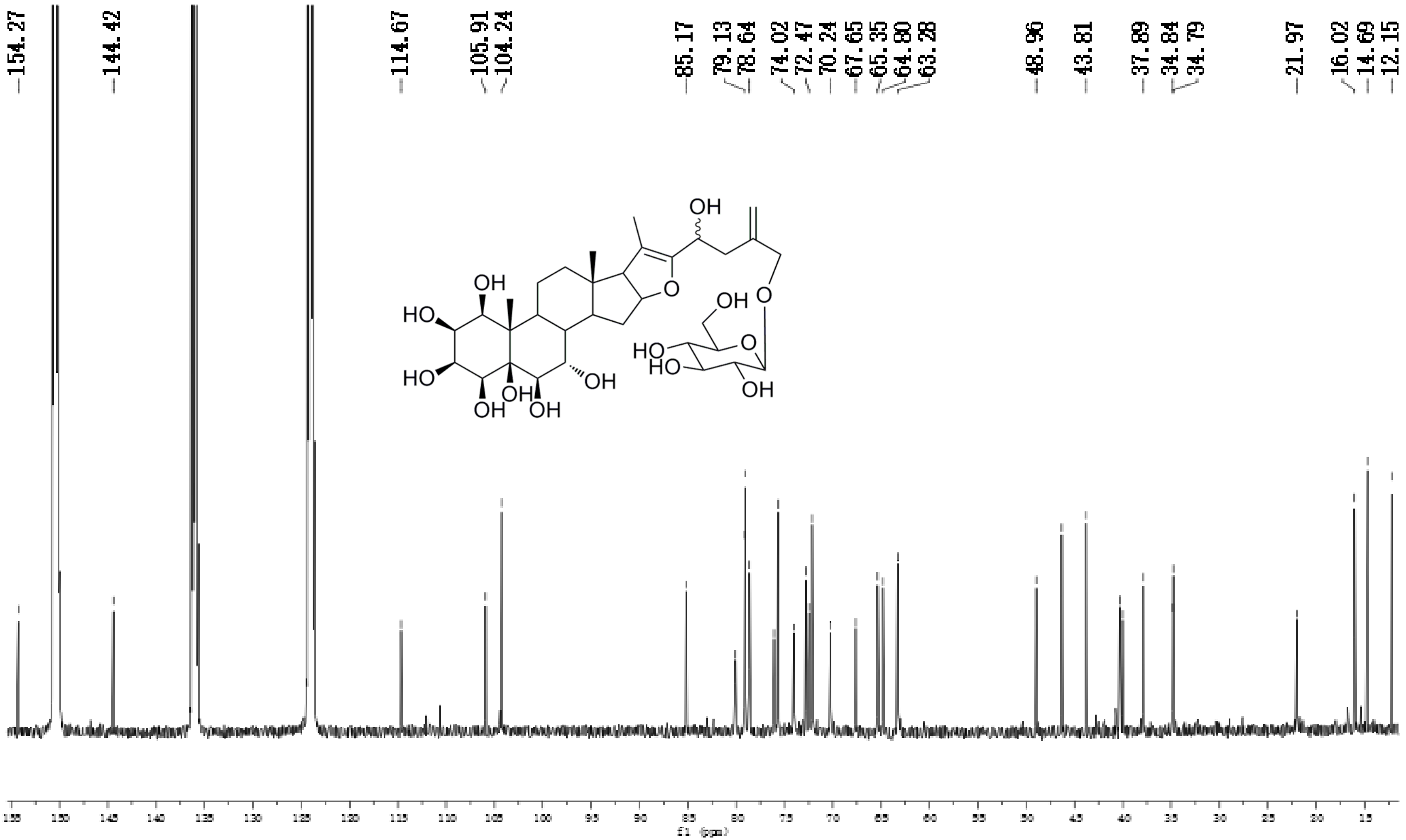

Figure S11. The ^{13}C-NMR spectrum of **2** (in C_5D_5N).

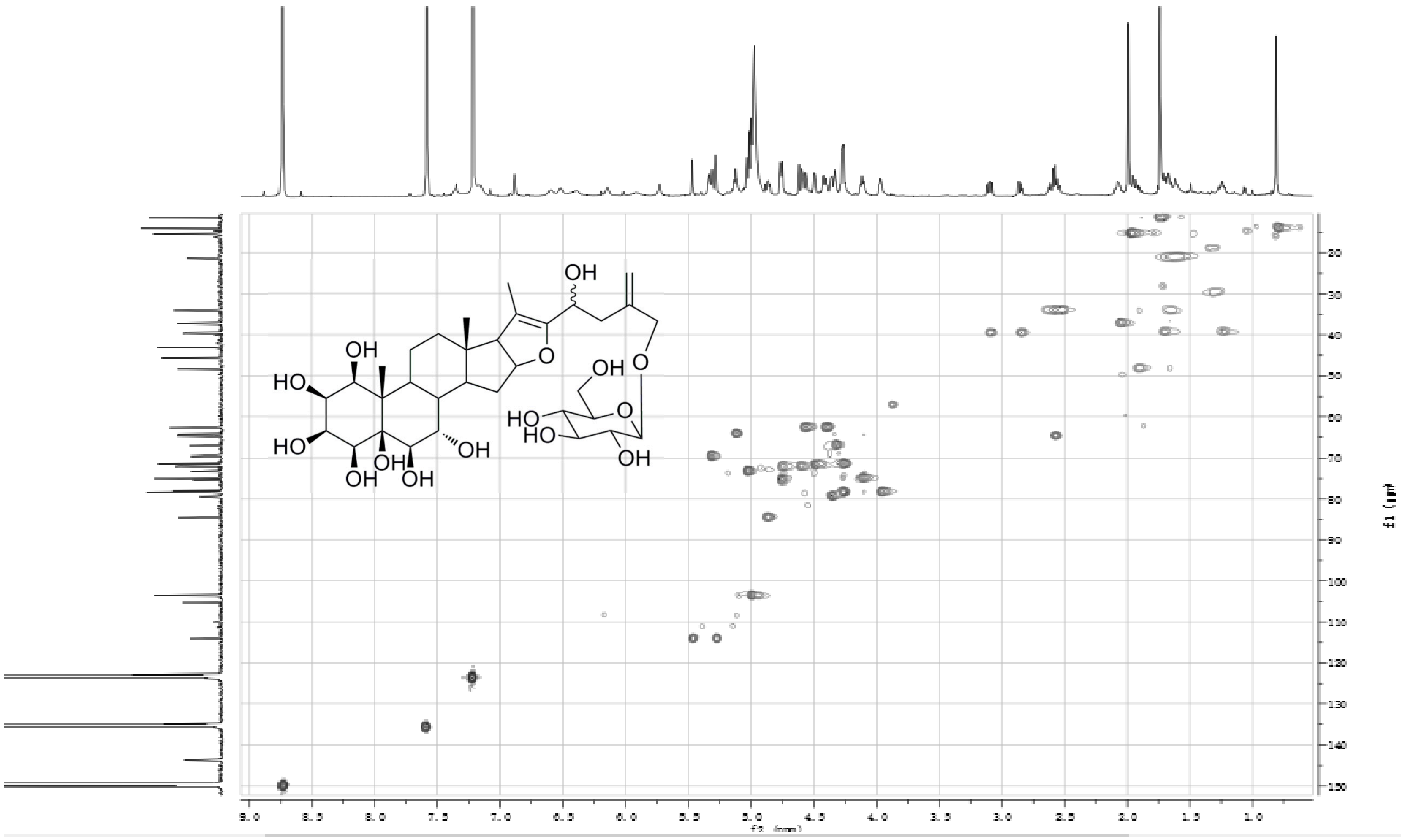

Figure S12. The HMQC spectrum of **2** (in C_5D_5N).

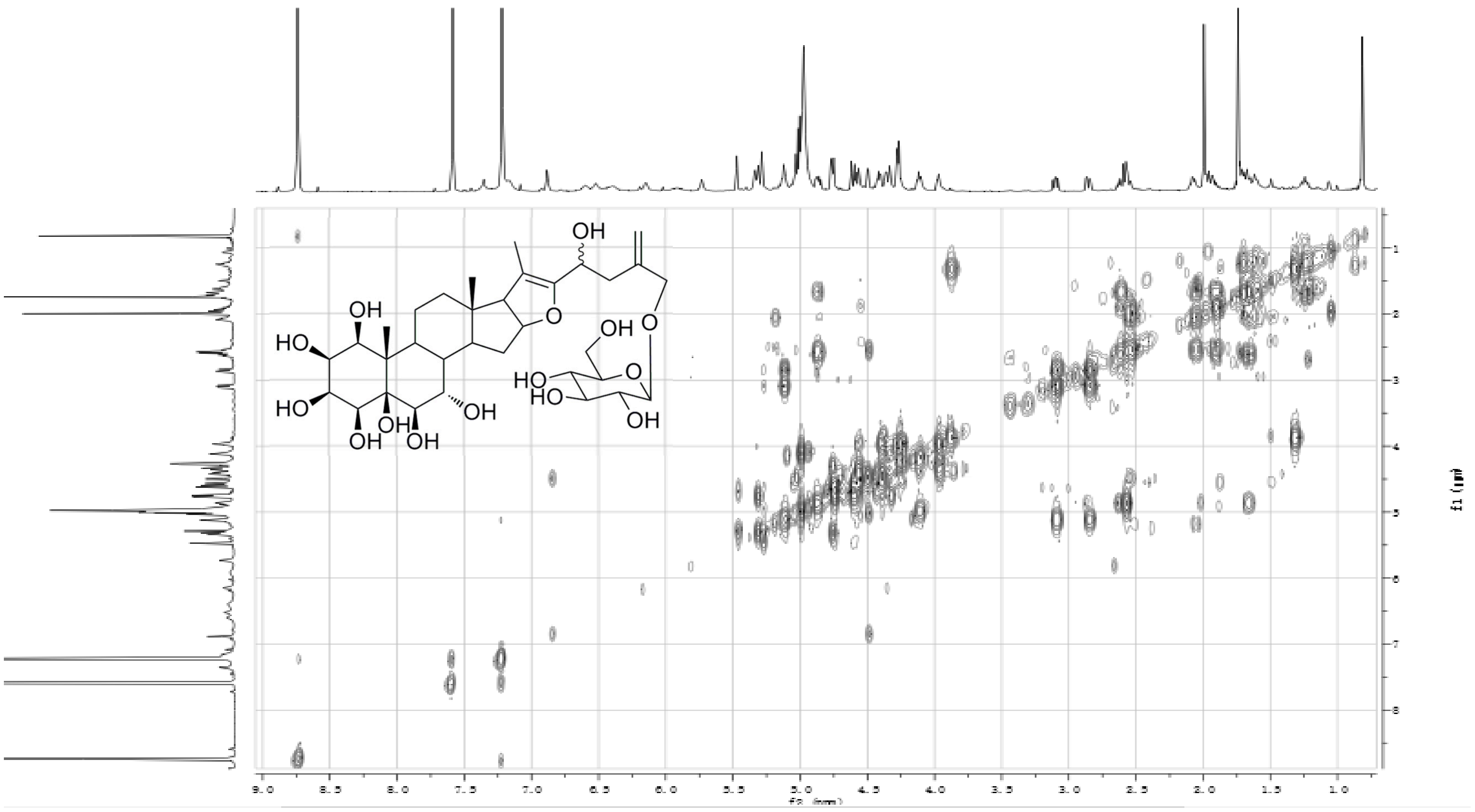

Figure S13. The ^{1}H-^{1}H COSY spectrum of **2** (in C_5D_5N).

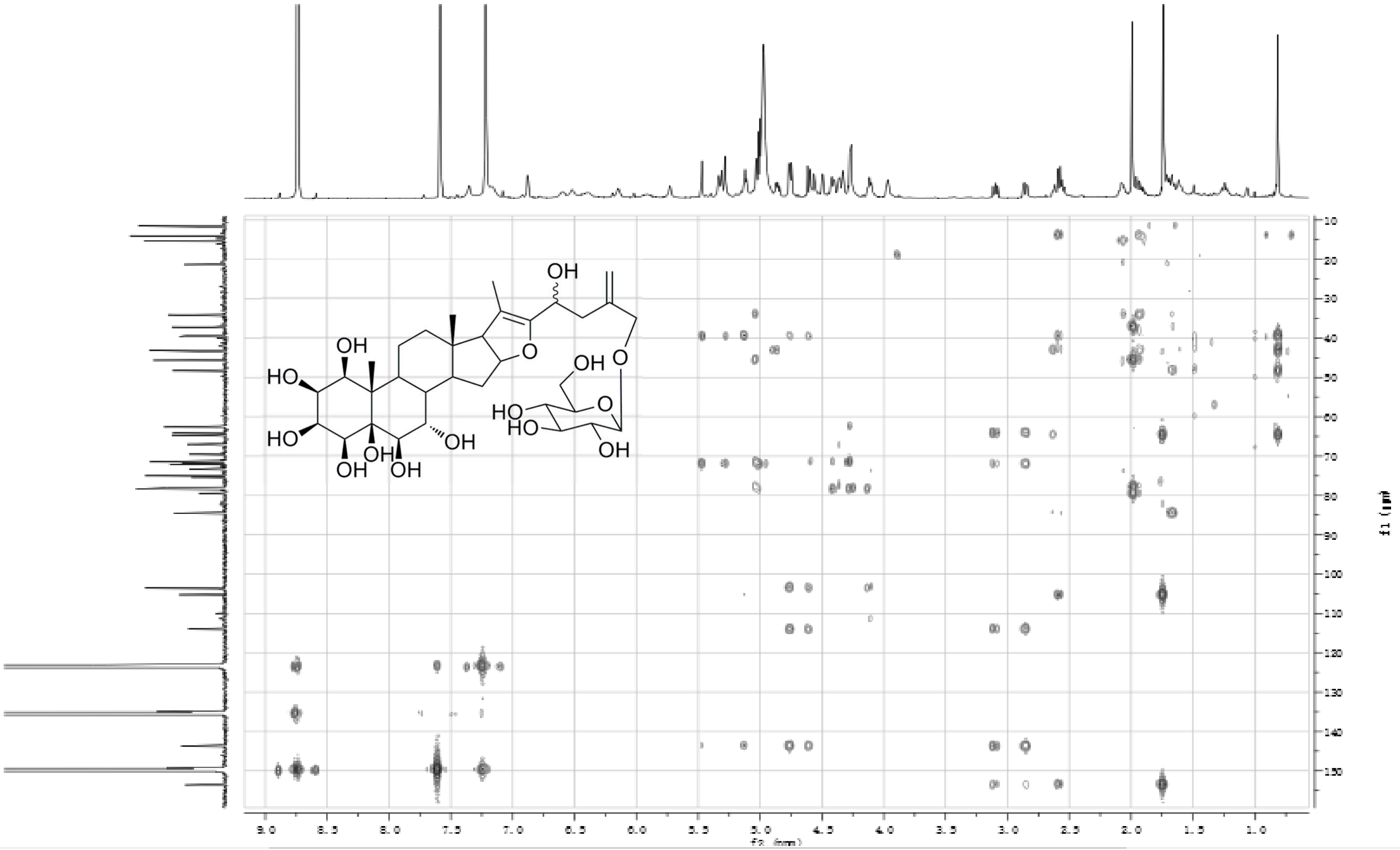

Figure S14. The HMBC spectrum of **2** (in C_5D_5N).

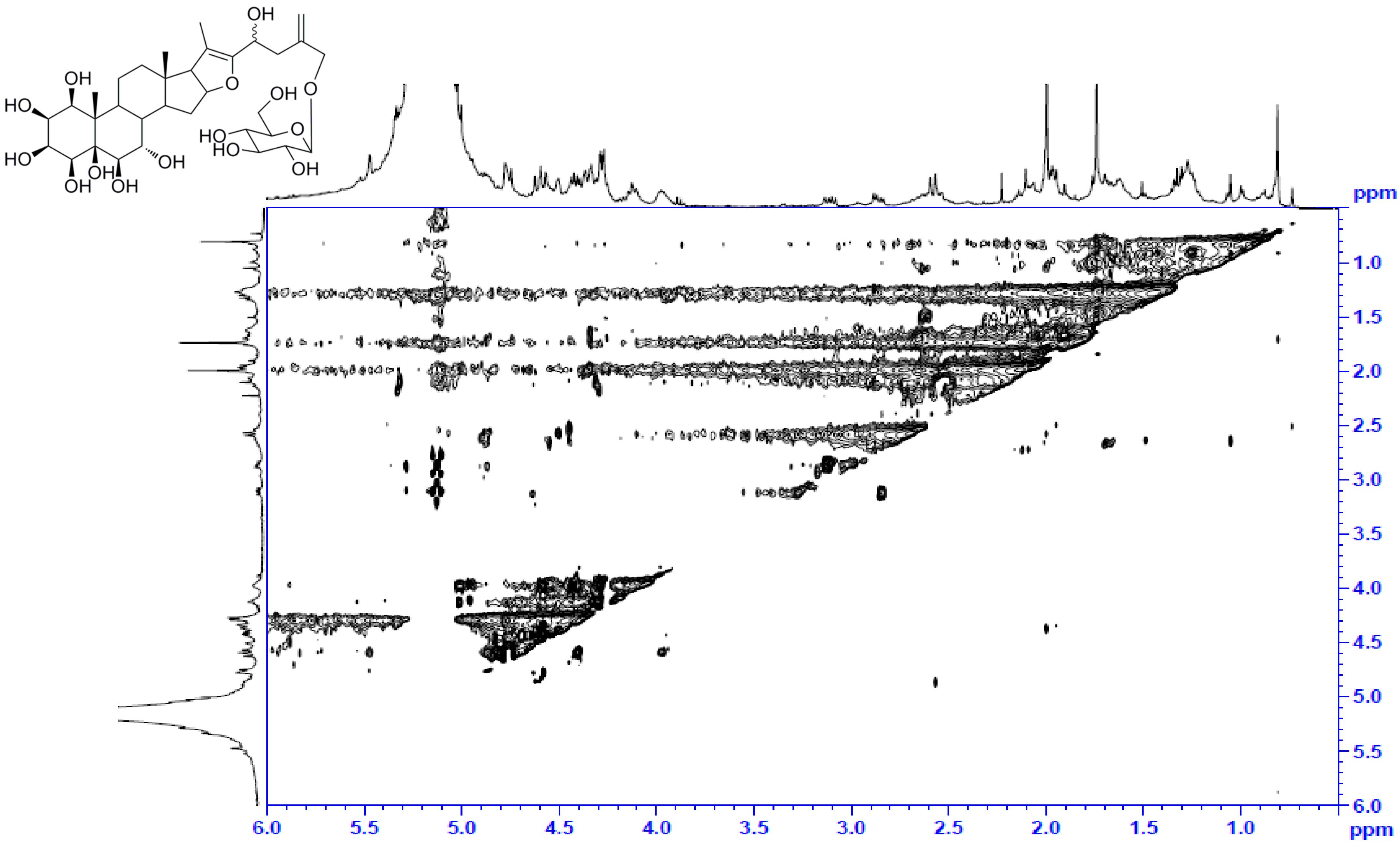

Figure S15. The NOESY spectrum of **2** (in C_5D_5N).

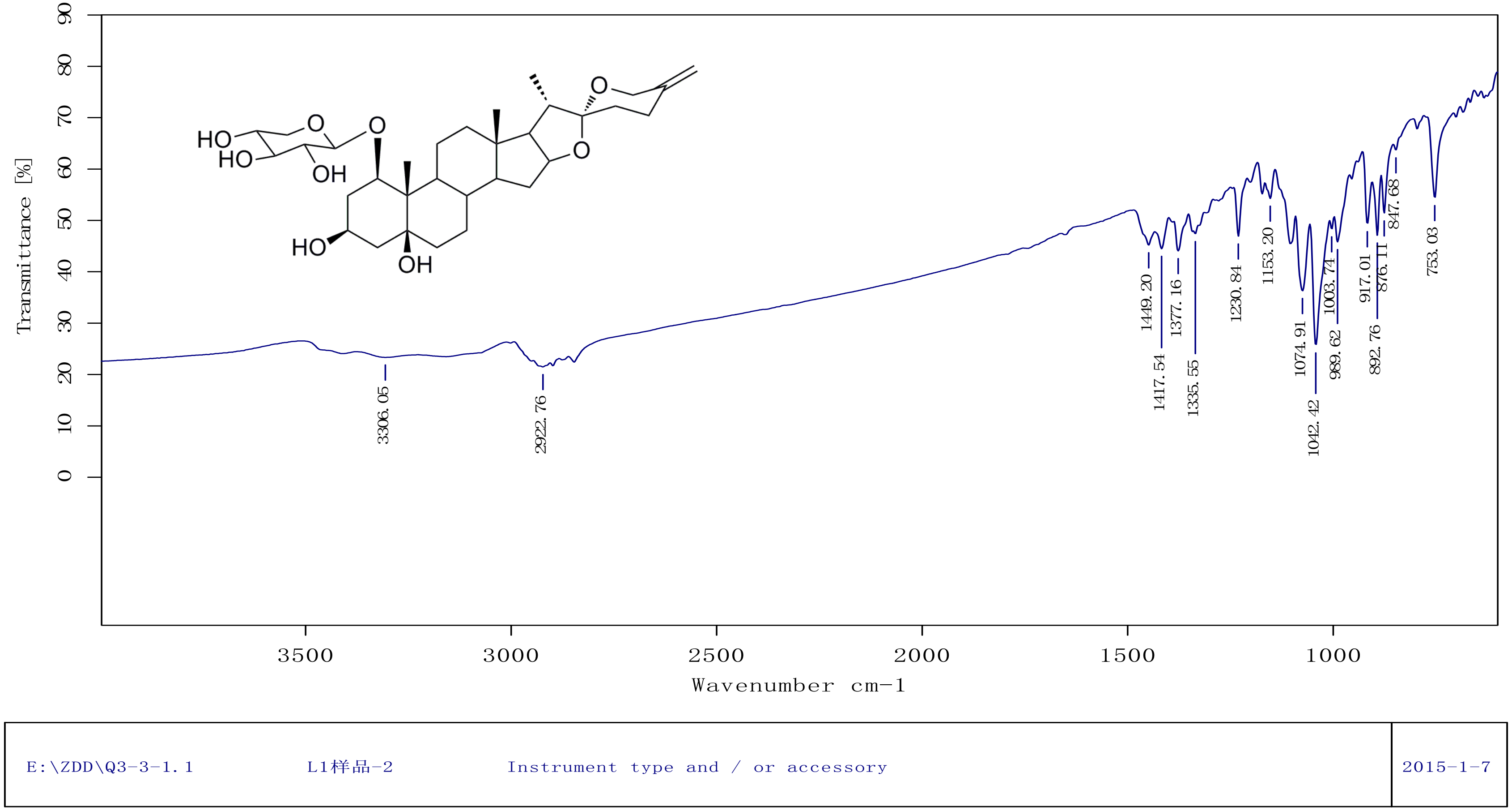

Page 1/1

Figure S16. The IR spectrum of **3** (in KBr).

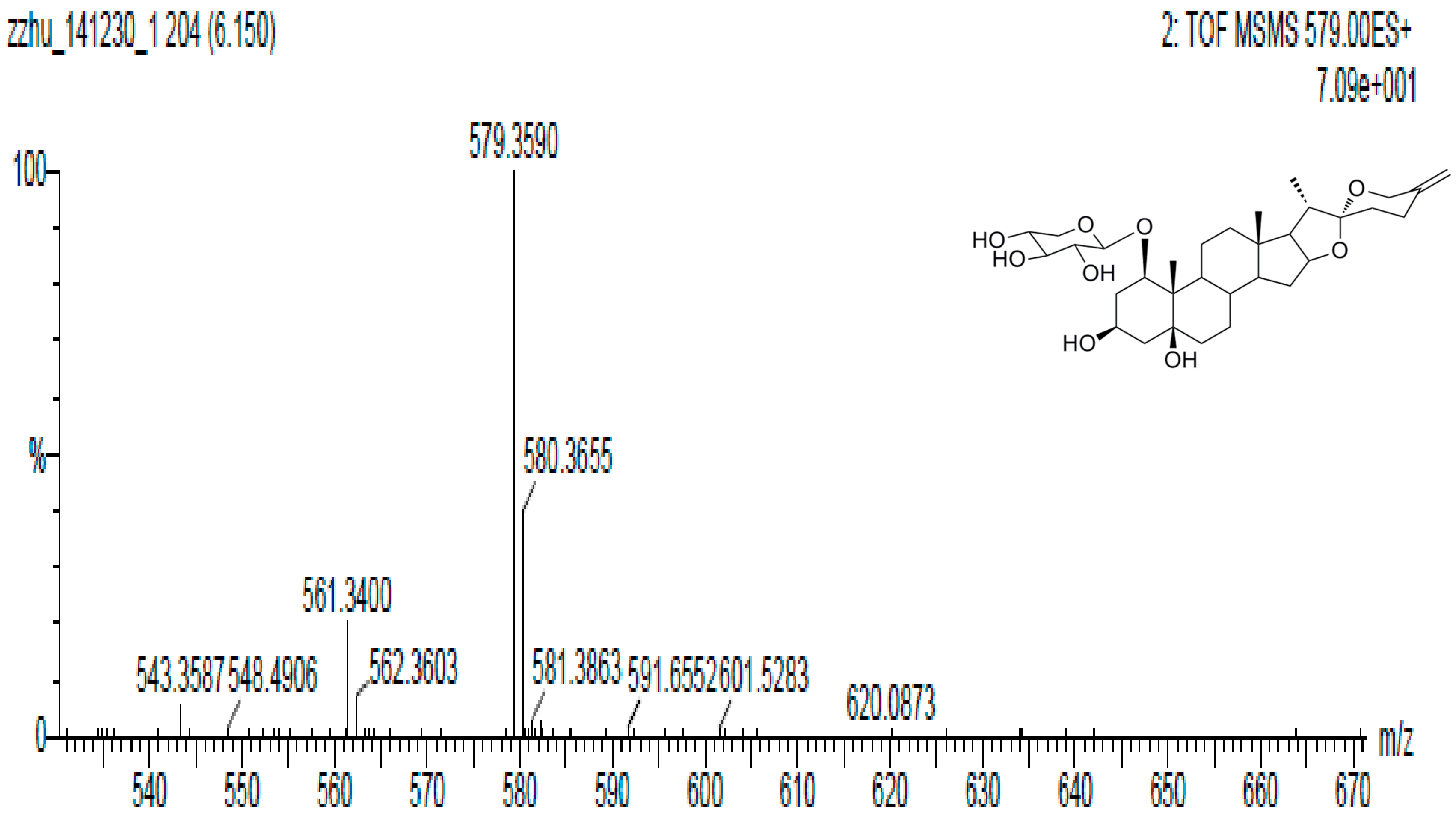

Figure S17. The HR-ESI-MS spectrum of **3** (in MeOH).

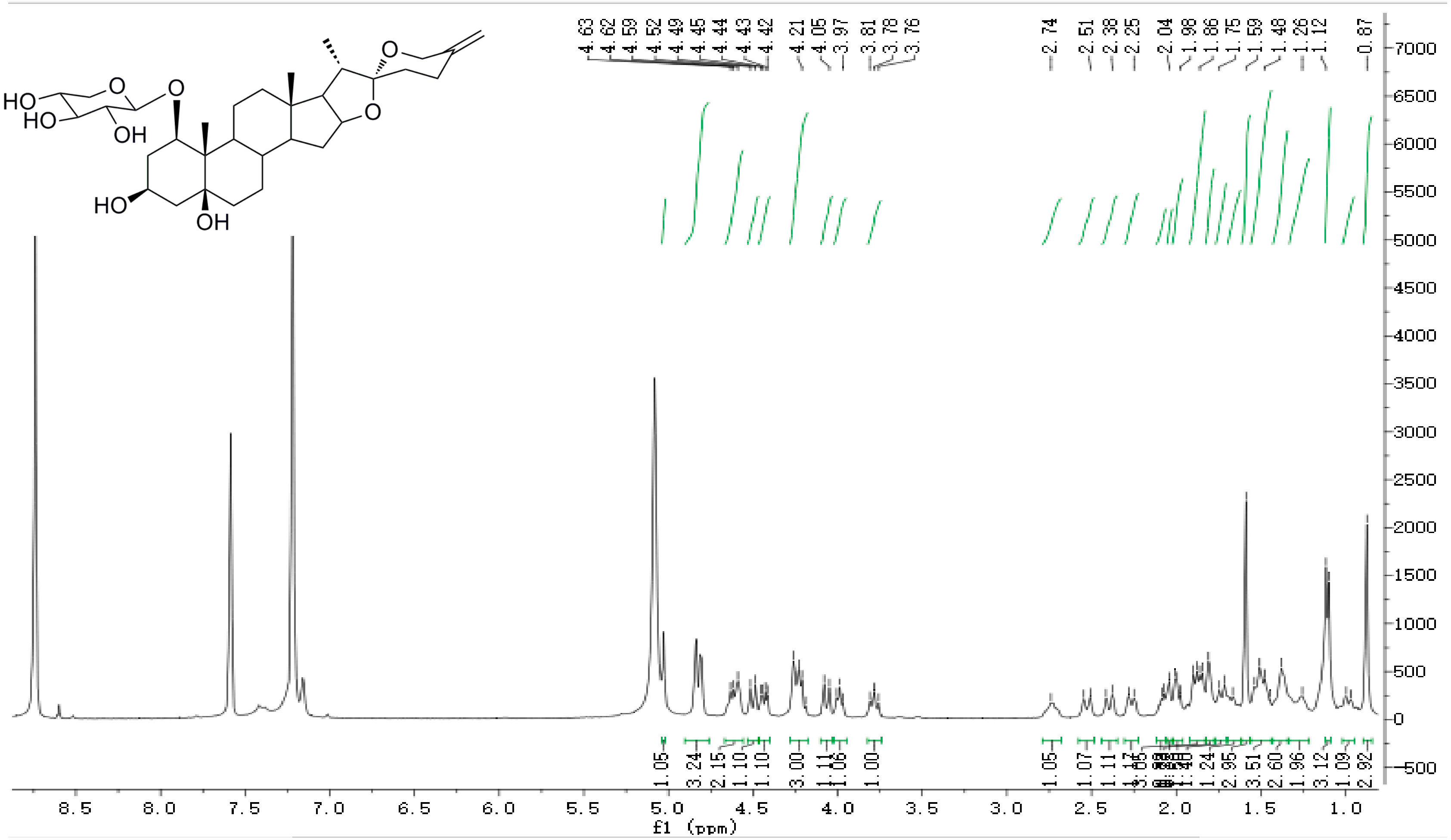

Figure S18. The ^{1}H-NMR spectrum of **3** (in C_5D_5N).

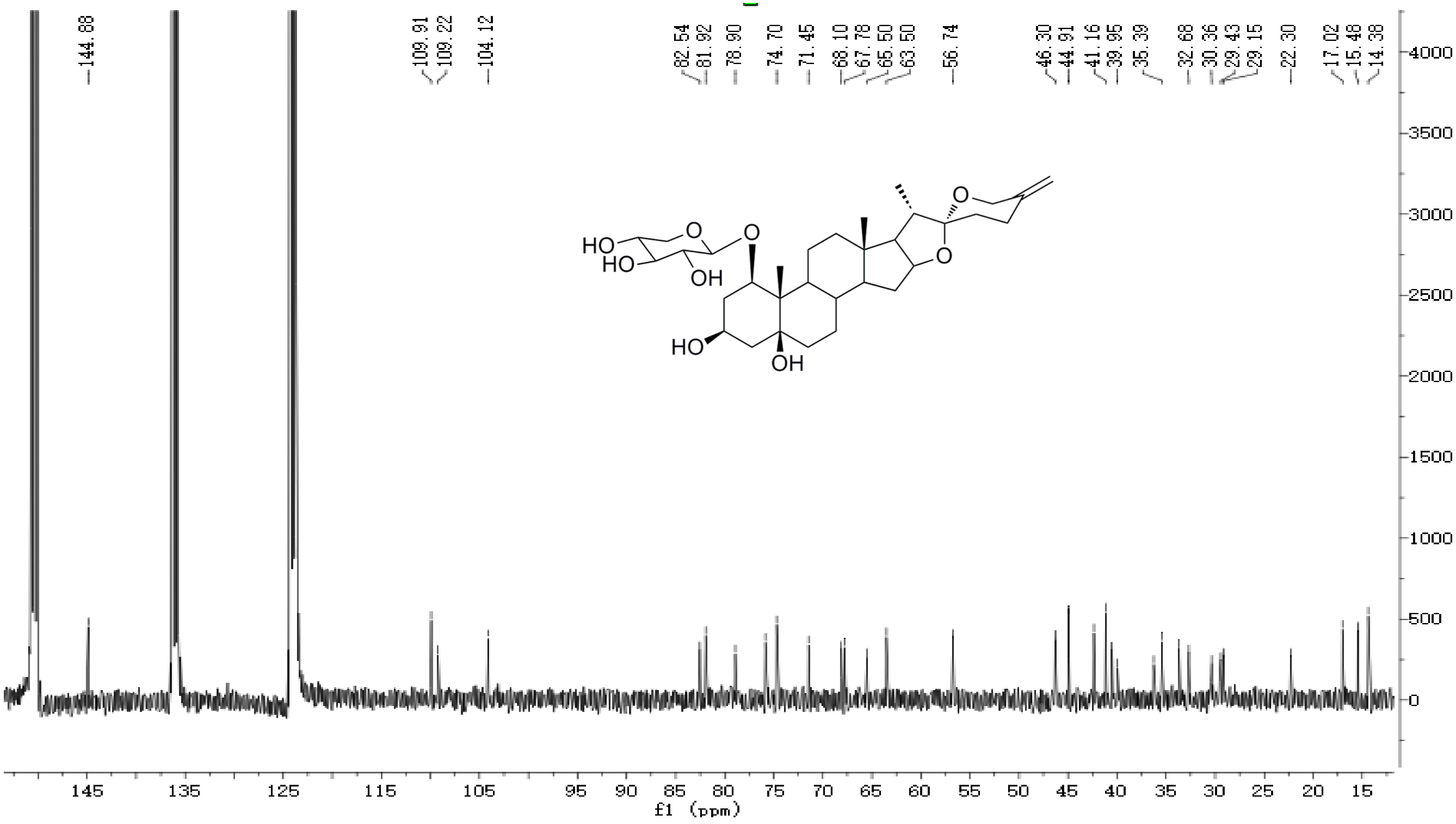

Figure S19. The ^{13}C-NMR spectrum of **3** (in C_5D_5N).

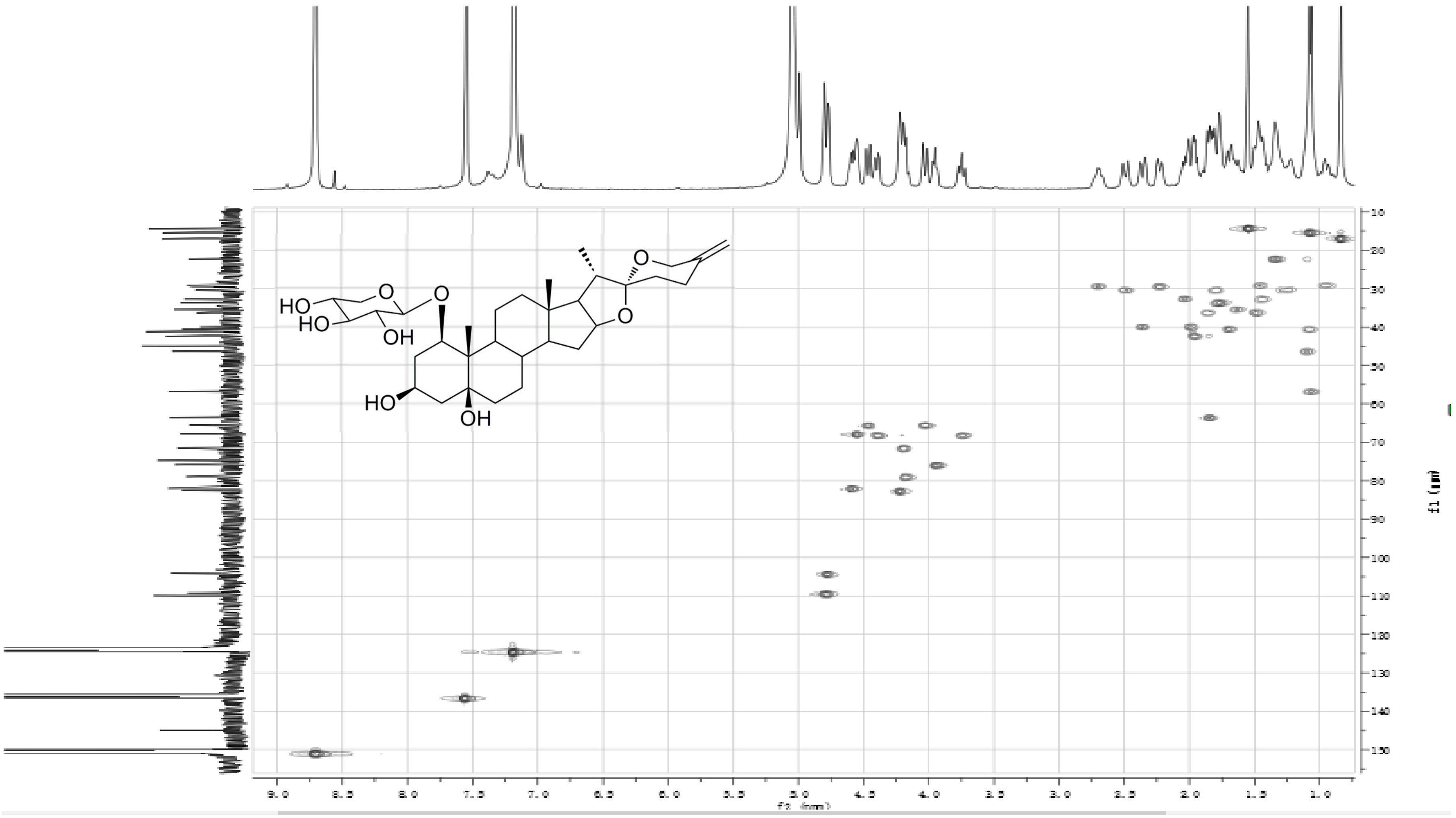

Figure S20. The HMQC spectrum of **3** (in C_5D_5N).

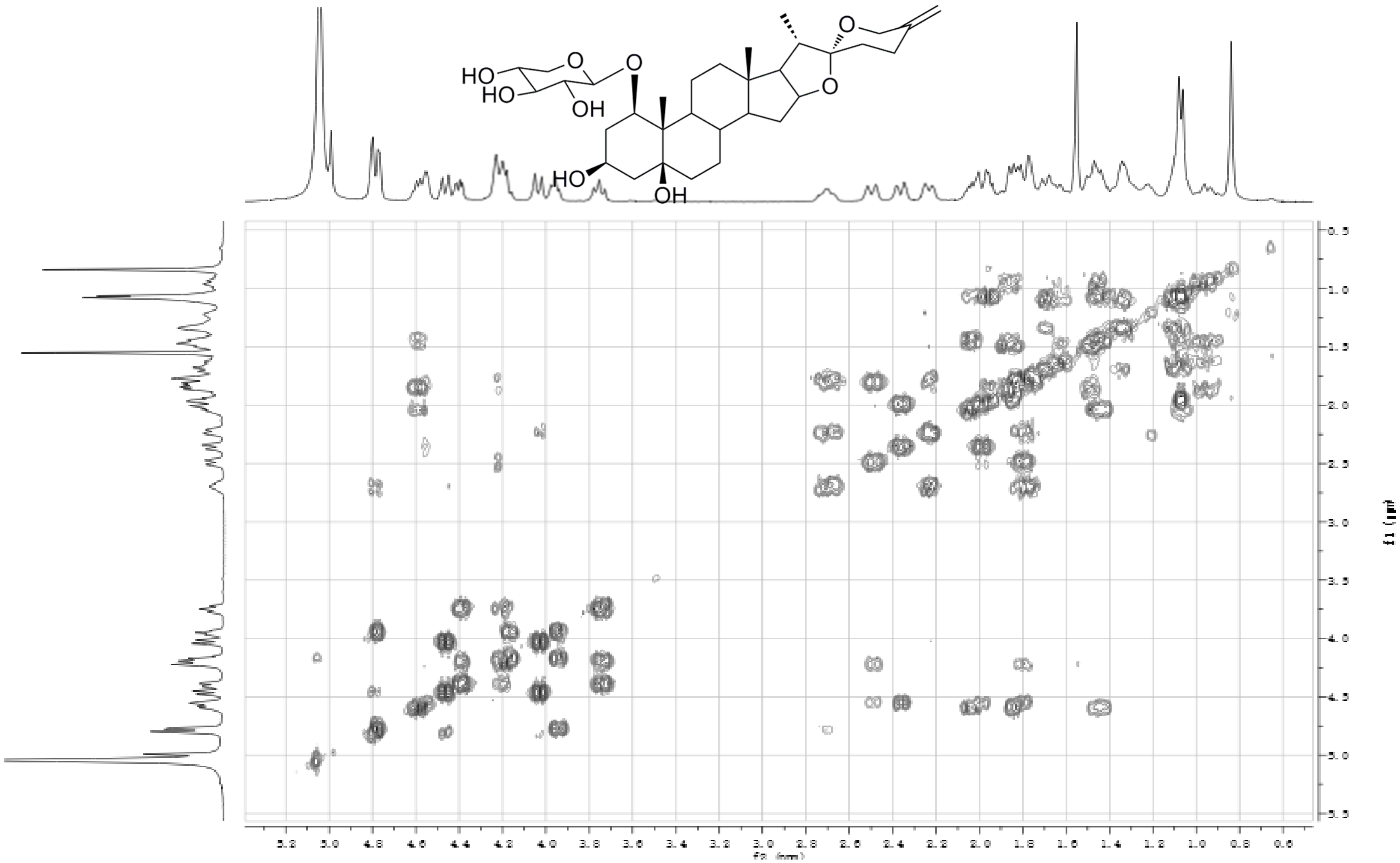

Figure S21. The ^{1}H-^{1}H COSY spectrum of **3** (in C_5D_5N).

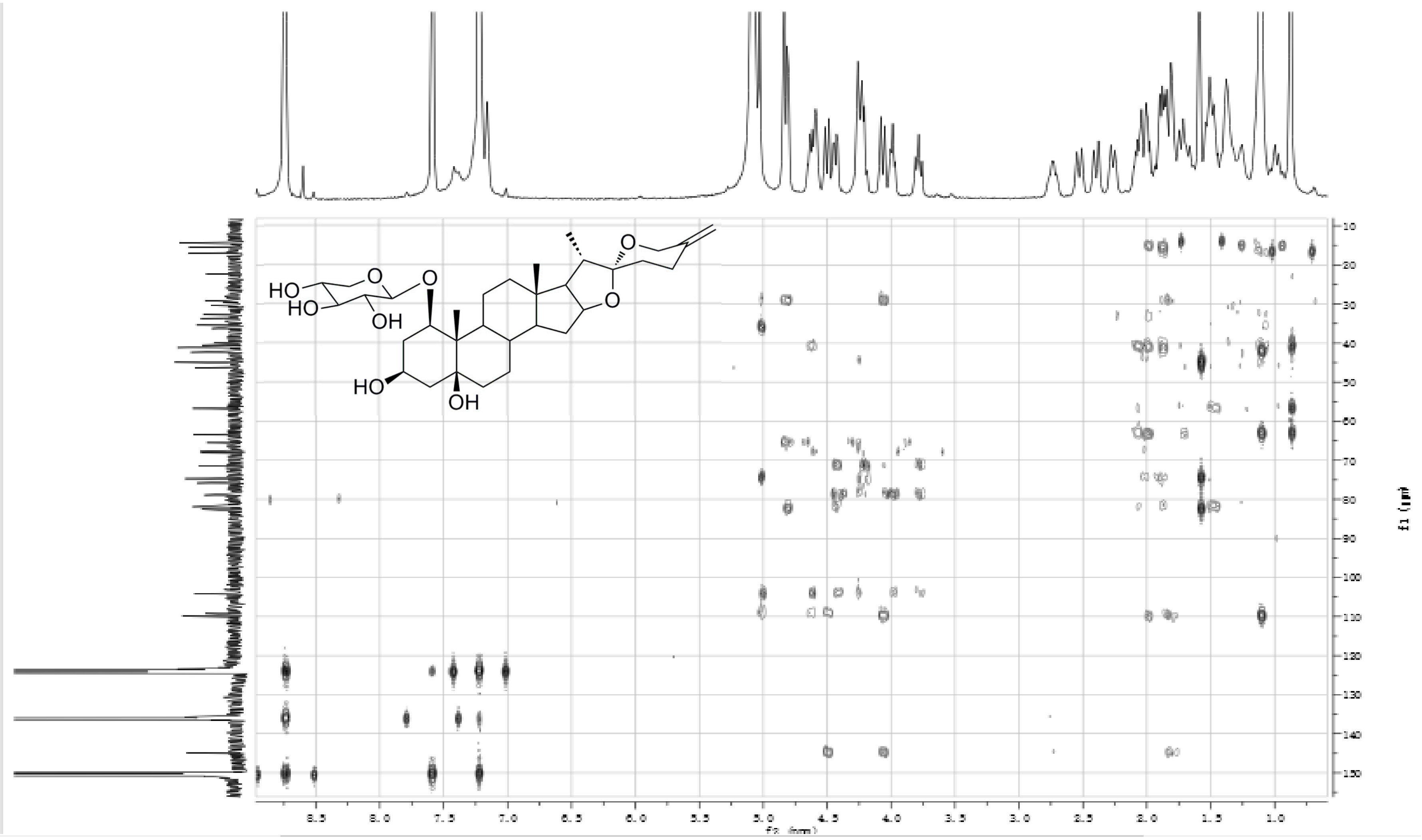

Figure S22. The HMBC spectrum of **3** (in C_5D_5N).

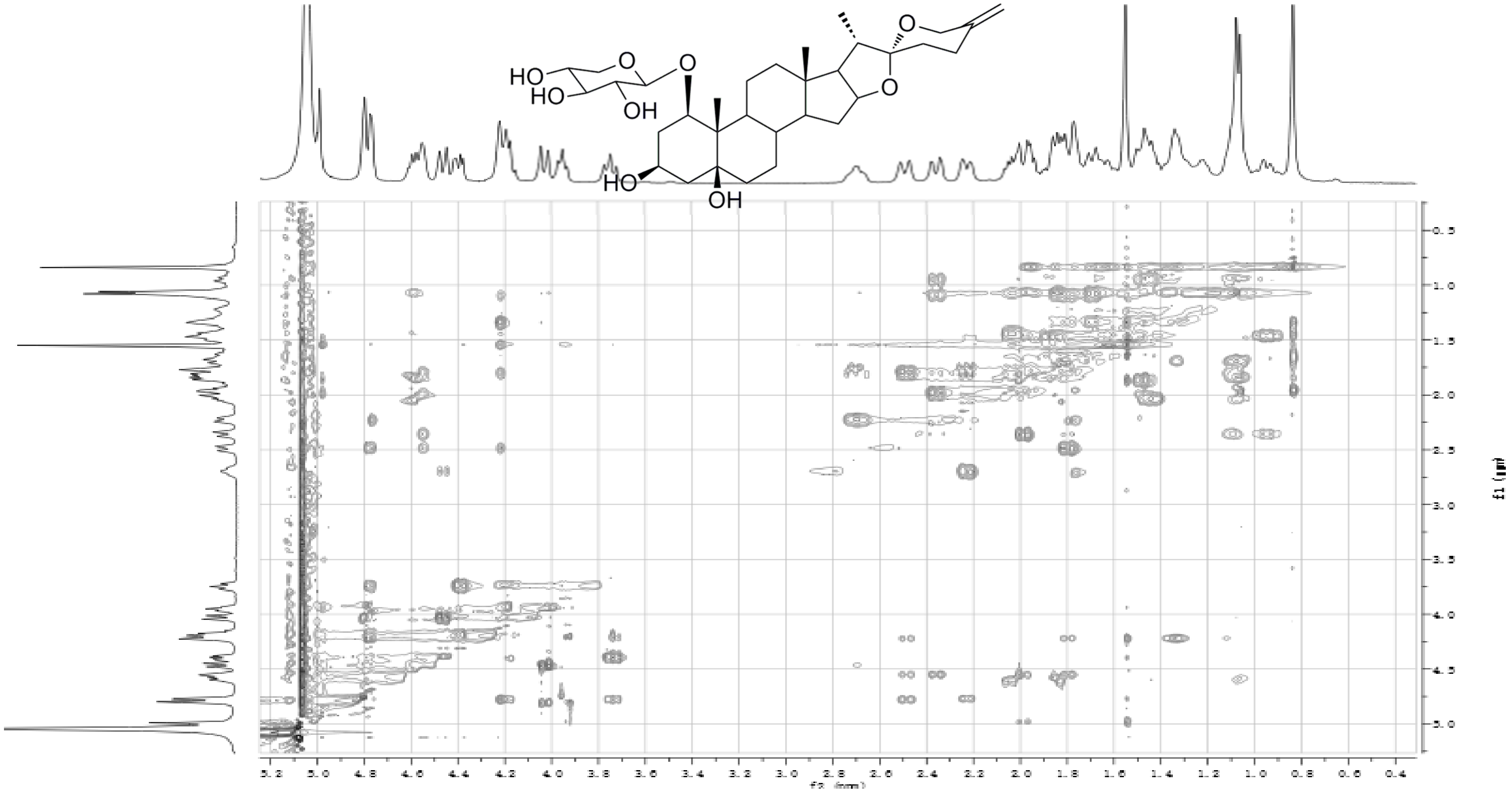

Figure S23. The NOESY spectrum of **3** (in C_5D_5N).
